# Supplementary material for: CARD11 mutation and HBZ expression induce lymphoproliferative disease and adult T-cell leukemia/lymphoma
Source: Commun Biol. 2022 Nov 29;5:1309. doi: 10.1038/s42003-022-04284-x (PMC9709164; doi:10.1038/s42003-022-04284-x)
Supplement: Supplementary file 2 — Supplementary Information [file 42003_2022_4284_MOESM2_ESM.pdf]

Supplementary Information

Summary of Supplementary Information

- **Supplementary Methods** explain our methods for western blotting, flow cytometry, histology, cell sorting, RNA extraction, cDNA library preparation for RNA sequencing, gene expression analysis, quantitative PCR, and transplantation assays.
- **Supplementary Table 1** lists gene sets that were enriched in GSEA analysis only in human acute-type ATL samples, and not in CARD11(E626K)$^{CD4\text{-Cre}}$;*HBZ* Tg mice.
- **Supplementary Table 2** lists reagents, genetically modified organisms and strains, primers for qPCR, cell lines, software, instrumentation, and source data used in this study.
- **Supplementary Figure 1** shows strategies for targeting mutant mice.
- **Supplementary Figure 2** shows western blot analysis of splenic CD4$^+$ T cells.
- **Supplementary Figure 3** shows pathological and flow cytometric analysis of the thymus.
- **Supplementary Figure 4** shows flow cytometric analysis of CD4$^+$ T cells, effector/memory T cells, regulatory T cells, and CD8$^+$ T cells in the bone marrow, spleen, and body.
- **Supplementary Figure 5** shows flow cytometric analysis of CD4$^+$ T cells, effector/memory T cells, and regulatory T cells in lymph nodes.
- **Supplementary Figure 6** shows pathological analysis of lymph nodes and lungs of mutant mice.
- **Supplementary Figure 7** shows pathological analysis of NOD/Shi-scid/IL-2Rγ^{null} (NOG) mice transplanted from CD4$^+$ T cells of mutant mice.
- **Supplementary Figure 8** shows global gene expression profiling of effector/memory T cells obtained from mutant mice.
- **Supplementary Figure 9** shows GSEA analysis of the canonical and non-canonical NF-κB signaling pathways.
- **Supplementary Figure 10** shows western blotting for the NF-κB pathway.
- **Supplementary Figure 11** shows GSEA analysis of HBZ target genes in mutant mice.
- **Supplementary Figure 12** shows GSEA analysis of genomic instability-related genes in mutant mice.
- **Supplementary Figure 13** shows gene expression analysis between CARD11(E626K)$^{CD4\text{-Cre}}$;*HBZ* Tg mice at 4–6 months and *HBZ* Tg mice at 8–12 months.

- **Supplementary Figure 14** shows GSEA analysis of IRF4 target genes in mice and human samples.
- **Supplementary Figure 15** shows GSEA analysis of MYC target genes and E2F target genes in mice and human samples.
- **Supplementary Figure 16** shows the experimental flowchart.
- **Supplementary Figure 17** shows uncropped blots of Figure 9c, d.
- **Supplementary Figure 18** shows uncropped blots of Supplementary Figure 2.
- **Supplementary Figure 19** shows uncropped blots of Supplementary Figure 10.
- **Supplementary Reference**

Supplementary Methods

Western blotting

Splenocytes from each mouse type (n = 2–3 for each type) were pooled between 8 months to 12 months after birth, then $CD4^+$ splenocytes were isolated using the mouse $CD4^+$ T-Cell Isolation Kit (Miltenyi Biotec, BG, Germany) and prepared for Western blotting. Total cell lysates were prepared as previously described[1]. Nuclear cell extracts were prepared using a Nuclear Extract Kit (Active Motif, CA, USA) or Nuclear/Cytosolic Fractionation Kit (AKR-172) (Cell Biolabs, CA, USA). Extracts were resolved by SDS-polyacrylamide gel electrophoresis, transferred to polyvinylidene difluoride nitrocellulose membranes, probed using the appropriate antibodies, and visualized by electrochemiluminescence (GE Healthcare, NJ, USA). Antibodies used and dilutions are listed in Supplementary Table 2. Bio-Rad Image Lab software was used to calculate band intensity.

Flow Cytometry

Tissues were processed into single-cell suspensions and treated with red blood cell lysis buffer, and cells were transferred into phosphate-buffered saline containing 2% fetal

bovine serum. The cells were blocked with Fc-block (BD Biosciences, CA, USA), stained with antibodies for 30 min at 4 °C, washed, and analyzed using a FACSCalibur Flow Cytometer (BD Biosciences), FACSCanto II Flow Cytometer (BD Biosciences), and FlowJo software (Tree Star, Inc., OR, USA). Antibodies used are listed in Supplementary Table 2.

Histology

Mouse organs were obtained from 6 and 12 months after birth (n = 5, in each type of mice) and fixed in 4% paraformaldehyde and paraffin embedded. Sections were stained with hematoxylin and eosin (HE). For immunohistochemistry, sections were stained with the appropriate primary and secondary antibodies. Antibodies used and dilutions are listed in Supplementary Table 2.

Quantitative histological analysis

The number of $CD44^+$, $FOXP3^+$, and $Ki67^+$ cells, together with the total nucleated cell number, was scored in 5 microscopic views from each of the 3 mouse types, and is presented as the proportion of $CD44^+$, $FOXP3^+$, or $Ki67^+$ cells relative to the total number of nucleated cells in each mice type in lymph nodes and lungs. As for lymph

nodes, the proportion of Ki-67$^+$ cells to CD44$^+$ T cells or Ki-67$^+$ cells to FOXP3$^+$ T cells was assessed using consecutive sections. Tukey's test after one-way ANOVA; *, **, *** represent p values less than 0.05, 0.01, and 0.001, respectively.

Cell sorting, RNA extraction, and cDNA library preparation for RNA sequencing

Splenocytes from each mouse type (n = 3–4 for each type) at 4 to 6 months after birth were stained with FITC Anti-CD4, PE Anti-CD8, APC Anti-CD25, and 7-AAD for sorting of CD4$^+$CD8$^-$CD25$^+$ Treg, and with FITC Anti-CD4, PE Anti-CD44, APC Anti-CD62L, and 7-AAD for sorting of CD4$^+$CD44$^+$CD62L$^-$ Tem, and then sorted with a FACSAria II cell sorter (BD Biosciences). Total RNA was extracted using Isogen (Nippon Gene, Tokyo, Japan), and cDNA was synthesized using the SMARTer Pico PCR cDNA Synthesis Kit (Clontech, CA, USA). Double-stranded cDNA was fragmented, and cDNA libraries were generated using the KAPA HyperPlus Library Preparation Kit (KAPA Biosystems, MA, USA) and FastGene Adapter Kit (Nippon Genetics, Tokyo, Japan). Sequencing was performed using NextSeq500 (Illumina, CA, USA) with a single-read sequencing length of 76 bp. Kallisto (version 0.43.1) was used for determining read counts and calculating transcripts per million [2].

Gene expression analysis

edgeR (version 3.12) and iDEP (version 0.92) were used for statistical analysis [3,4]. R (version 4.0) and the Heatmap.2 function from the gplots package were used for creating heatmaps. Gene set enrichment analysis (GSEA) was performed using Molecular Signatures Database (MSigDB)–curated gene sets (KEGG, HALLMARK, GO biological process, and REACTOME gene sets) and Lymphoma/Leukemia Molecular Profiling Project (LLMPP)–curated gene sets[5-7]. False discovery rate (FDR) q-values ≤0.25 were considered significant. For differential expressional analysis, differentially expressed genes between sample groups were identified by iDEP using cut-offs of fold change (FC) >1.2 and a FDR of <0.1[4]. Functional annotation of identified genes was carried out in Enrichr[8]. Batch correction between different datasets was performed using ComBat-seq[9]. The reagents, data sets, and software used are listed in Supplementary Table 2.

Isolation of RNA, reverse transcription, and quantitative PCR (qPCR)

RNAs were extracted using the RNeasy Micro Kit (QIAGEN, Hilden, Germany). Reverse transcription was carried out with 20 ng total RNA using QuantiTect Reverse TranscriptionKit (QIAGEN). Quantitative PCR was performed on a LightCycler 480

(Roche Applied Science) with a universal protocol using SYBR Green. Gene-specific primer sets are listed in Supplementary Table 2. Gene expression levels were normalized to *Gapdh*.

Transplantation assays

To determine the hallmark of malignant transformation, we transplanted mutant CD4$^+$ T cells into NOD/Shi-scid/IL-2Rγ^{null} (NOG) mice and assessed their transplantability and tumorigenicity. A total of 1–2 x 10^6 CD4$^+$ splenic T cells from WT, CARD11(E626K)$^{\mathit{CD4}\text{-Cre}}$, *HBZ* Tg, and CARD11(E626K)$^{\mathit{CD4}\text{-Cre}}$;*HBZ* Tg mice were isolated by magnetic activated cell sorting (MACS) and transplanted intravenously to 3 NOG mice per donor mouse type. Recipients were sacrificed at 18 weeks after transplantation, and assessed by pathology of the spleen and lymph nodes (if lymph nodes were visible).

Supplementary Table 1. Gene sets which were only enriched in human ATL acute type samples, but not in CARD11(E626K)CD4-Cre;HBZ Tg mice.

pathway name	human comparison	human NES	human FDR	mouse comparison	mouse NES	mouse FDR
KEGG_ASCORBATE_AND_ALDARATE_METABOLISM	Acute ATL vs. Healthy	1.869	0.010	Compound vs. WT	0.879	0.677
KEGG_LINOLEIC_ACID_METABOLISM	Acute ATL vs. Healthy	-1.700	0.051	Compound vs. WT	-0.737	0.994
KEGG_PORPHYRIN_AND_CHLOROPHYLL_METABOLISM	Acute ATL vs. Healthy	1.748	0.054	Compound vs. WT	1.136	0.338
KEGG_RIBOFLAVIN_METABOLISM	Acute ATL vs. Healthy	1.552	0.151	Compound vs. WT	-0.824	0.987
KEGG_REGULATION_OF_AUTOPHAGY	Acute ATL vs. Healthy	1.564	0.158	Compound vs. WT	0.984	0.521
KEGG_ALANINE_ASPARTATE_AND_GLUTAMATE_METABOLISM	Acute ATL vs. Healthy	1.477	0.195	Compound vs. WT	-0.904	1.000
KEGG_PENTOSE_AND_GLUCURONATE_INTERCONVERSIONS	Acute ATL vs. Healthy	1.632	0.200	Compound vs. WT	1.101	0.392
KEGG_BIOSYNTHESIS_OF_UNSATURATED_FATTY_ACIDS	Acute ATL vs. Healthy	1.458	0.202	Compound vs. WT	1.055	0.442
KEGG_GLYCEROLIPID_METABOLISM	Acute ATL vs. Healthy	1.444	0.203	Compound vs. WT	-0.911	1.000
KEGG_CARDIAC_MUSCLE_CONTRACTION	Acute ATL vs. Healthy	1.432	0.212	Compound vs. WT	0.953	0.554
KEGG_MELANOMA	Acute ATL vs. Healthy	1.383	0.213	Compound vs. WT	1.045	0.449
KEGG_BUTANOATE_METABOLISM	Acute ATL vs. Healthy	1.427	0.215	Compound vs. WT	1.206	0.261
KEGG_ADHERENS_JUNCTION	Acute ATL vs. Healthy	1.387	0.216	Compound vs. WT	0.766	0.852
**KEGG_NOTCH_SIGNALING_PATHWAY**	**Acute ATL vs. Healthy**	**1.399**	**0.224**	**Compound vs. WT**	**0.917**	**0.612**
KEGG_EPITHELIAL_CELL_SIGNALING_IN_HELICOBACTER_PYLORI_INFECTION	Acute ATL vs. Healthy	1.404	0.226	Compound vs. WT	1.022	0.471
KEGG_BETA_ALANINE_METABOLISM	Acute ATL vs. Healthy	1.350	0.239	Compound vs. WT	1.075	0.416
KEGG_N_GLYCAN_BIOSYNTHESIS	Acute ATL vs. Healthy	1.338	0.245	Compound vs. WT	1.093	0.399

Genesets with human FDR<0.25 and mouse FDR>0.25 are listed by decreasing order of humn FDR value. Metabolism-related pathways are colored. KEGG_NOTCH_SIGNALING_PATHWAY are displayed in bold.

Supplementary Table 2. The reagents, genetically modified organisms and strains, cell lines, software, instrumentation, and source data.

REAGENT or RESOURCE		SOURCE	IDENTIFIER
Experimental Models: Organisms/Strains			
Mouse: C57/BL/6 *CD4*-Cre Tg		The Jackson lab	#022071
Mouse: C57/BL/6 CARD11(E626K)^{stopFL}		This paper	N/A
Mouse: C57/BL/6 CARD11(E626K)$^{CD4\text{-}Cre}$		This paper	N/A
Mouse: C57/BL/6 *HBZ* Tg		This paper	N/A
Mouse: C57/BL/6 CARD11(E626K)$^{CD4\text{-}Cre}$;*HBZ* Tg		This paper	N/A
Experimental Models: Organisms/Cell line			
S1T		Dr. Yoshimitsu and Dr. Ishitsuka (Kagoshima Univ.)	DOI: 10.1128/JVI.65.12.6892-6899.1991
HPB-ATL2		Dr. Morikawa and Dr. Matsuoka (Kumamoto Univ.)	DOI: 10.1002/ijc.2910380218
ATL-43		Dr. Maeda and Dr. Matsuoka (Kumamoto Univ.)	DOI: 10.3389/fmicb.2020.00356.
＊These cell lines used tested negative for mycoplasma contamination using a PCR Mycoplasma test kit (VWR 10181-030).			
Oligonucleotides			
Forward primer for *Card11* E626K: 5'-AAGACCATCTGGGTGGACGA-3'		This paper	N/A
Forward primer for wild-type *Card11*: 5'-CCGCCTCGGAGTATTTTCCA-3'		This paper	N/A
Reverse primer for mutant and wild-type *Card11*: 5'-		This paper	N/A
Forward primer for *Batf3*: 5'-AACCAGCCGCAGAGCCC-3'		This paper	N/A
Reverse primer for *Batf3*: 5'-GAGCTGCAACCCGGTTTTTC-3'		This paper	N/A
cDNA sequences			
Card11 E626K		This paper	N/A
5'-<br>CCAGGAGGAGGGCCAGCTATGGATGACTACATGGAGACGCTGAAGGATGAAGAGGAGGCCCTATGGGATAACGTGGAATGCAACCGGCACATGCTGAGCCGTTACATCAACCCCGCCAAGCTCACCCCCTACCTGCGCCAGTGCAAGGTCATCGATGAGCAAGATG<br>AAGACGAGGTGCTCAATGCGCCCATGCTGCCGTCCAAGATCAACCGTGCAGGCCGATTGTTGGACATTCTTCACACCAAGGGACAAAGGGGCTATGTGGTCTTCCTGGAGAGCCTGGAGTTTTACTACCCAGAACTTTACAAACTGGTGACTGGAAAGGAACCCACC<br>CGGAGATTCTCCACCATTGTGGTGGAGGAAGGCCATGAGGGCCTCACACACTTCCTGATGAACGAGGTCATCAAACTGCAGCAGCAAGTGAAAGCCAAGGACCTTCAGCGCTGTGAGCTGCTGGCCAAGTCCCGGCAACTGGAGGATGAGAAGAAGCAGCTGAGCC<br>TGATACGGGTGGAGCTGCTGACCTTCCAGGAGCGATACTACAAGATGAAGGAGGAGCGGGACAGCTACAATGACGAGCTCGTCAAGGTCAAGGACGACAACTACAACTTAGCCATGCGCTACGCCCAGCTCAGTGAGGAGAAAAACATGGCGGTGATGAGGAGCC<br>GCGACCTCCAACTCGAGATCGACCAGCTCAAACACCGACTGAACAAGATGGAGGAGGAATGCAAGCTGGAGAGAAATCAGTCCCTCAAGCTCAAGAATGACATCGAGAACCGGCCCAGGAAGGAGCAGGTCCTGGAGCTGGAGCGGGAGAATGAGATGCTGAAGA<br>CGAAAATTCAGGAGCTGCAGTCCATCATCCAGGCTGGCAAGCGCAGCCTCCCTGACTCAGACAAGGCCATCTTGGACATCCTGGAACATGACCGGAAGGAGGCGCTAGAGGACCGGCAGGAACTGGTCAACAAAATTTACAACCTACAAGAGGAAGTCCGCCAGGC<br>GGAGGAGCTGCGGGATAAGTACCTGGAGGAGAAGGAAGACCTGGAACTCAAGTGTTCAACCCTGGGGAAGGACTGTGAAATGTACAAGCACCGCATGAACACAGTTATGCTGCAGCTGGAGGAGGTGGAGCGGGAGCGGGACCAGGCCTTCCACTCCCGAGATGA<br>GGCACAGACACAGTACTCACAGTGCTTAATCGAGAAGGACAAGTACCGGAAGCAGATCCGGGAGCTGGAGGAGAAGAACGATGAGATGCGTATTGAGATGGTGAGGAGGGAGGCCTGTATTGTCAACCTGGAAAGCAAGCTCCGGCGCCTGTCCAAGGACAACGG<br>CAGCCTCGACCAGAGTCTGCCTAGACACCTTCCAGCCACCATCATCTCACAGAACCTTGGAGACACCAGCCCCAGGACCAATGGCCAGGAAGCTGATGATTCTTCAACCTCAGAAGAGTCTCCCGAAGACAGCAAGTACTTTCTGCCTTACCACCCACCCCGGCGCC<br>GGATGAACCTAAAGGGCATCCAGCTGCAGAGAGCCAAATCCCCCATCAGCATGAAGCAAGCATCTGAGTTTCAAGTCAAGGGGCACGAAGAGGATTTCACAGACGGCAGCCCCAGTTCCTCCCGCTCGCTGCCTGTCACCAGCTCTTTCTCCAAGATGCAACCCCAT<br>CGGAGCCGCAGCAGCATCATGTCAATCACGGCAGAGCCCCCGGGAAATGACTCCATAGTCAGACGCTGTAAGGAAGATGCGCCACACCGGAGCACGGTGGAAGAAGACAACGATAGCTGTGGGTTTGATGCCTTAGACCTTGACGATGAAAATCACGAACGTTATT<br>CCTTTGGACCTCCCTCCATCCACTCCTCCTCCTCTTCACACCAGTCA<u>**aAG**</u>GGACTGGATGCCTACGACCTGGAGCAGGTCAACCTCATGTTACGAAAGTTCTCTTTGGAAAGGCCCTTCCGGCCATCGGTCACATCTGGGGGTCACGTGCGGGGCACCGGGCCCTTG<br>GTCCAGCACACAACTCTGAATGGCGATGGGCTCATCACGCAGCTCACCCTTCTGGGCGGCAATGCACGCGGGAGCTTCATTCACTCTGTCAAGCCAGGCTCACTGGCTGAGAGGGCCGGACTGCGTGAGGGCCACCAACTCCTGCTGCTGGAAGGTTGCATCCGAG<br>GCGAAAGGCAGAGCGTTCCACTGGATGCGTGCACAAAAGAAGAGGCCCGTTGGACCATCCAGAGGTGCAGTGGCCTCATCACTCTGCATTACAAGGTCAACCATGAAGGATACCGGAAGCTGCTGAAGGAGATGGAGGATGGTCTGATCACATCAGGGGACTCGTT<br>CTATATCCGCCTGAACCTGAACATCTCCAGCCAGCTGGATGCCTGCTCCATGTCCCTCAAGTGTGACGACGTGGTGCATGTCCTAGACACCATGTACCAGGACAGGCACGAGTGGCTGTGTGCACGAGTCGACCCCTTCACTGACCAAGACCTGGACACGGGCACCA<br>TCCCCAGCTACAGCCGGGCTCAACAGCTTCTCCTGGTGAAGCTCCAGCGGTTGGTTCACAGAGGCAACCGGGAAGAGGCAGACAGCGCTCACCACACCCTGCGCAGCCTCCGGAACACCCTGCAGCCCGAAGAGATGCTTTCGACGAGCGACCCCCGAGTCAGCCC<br>CCGCCTCTCCAGAGCGAGTTTCTTCTTTGGCCAGCTCCTGCAGTTTGTCAGCCGGTCAGAAAACAAGTACAAAAGAATGAACAGCAATGAGCGCGTGAGAATCATCTCTGGGAGTCCCCTGGGGAGCCTCTCCCGGTCCTCGCTGGATGCCACCAAACTCCTGACCG<br>AGAAGCATGAAGAACTGGATCCTGAGAATGAGCTCAGCCGGAACCTCACCCTGATCCCTTACAGCCTGGTGCGCGCTTTCCACTGTGAGCGCCGCAGGCCTGTGCTCTTCACGCCCACCATGCTGGCCAAGACATTGGTGCAGAAGCTGCTCAACTCAGGGGGTGCC<br>ATGGAGTTCACCATCTGCAAGTCAGATATTGTCACAAGAGATGAGTTCCTCCGAAAGCAGAAGACAGAGACCATCATCTACTCCCGGGAAAAGAAC1CCCAACACCTTTGAATGCATCGTCCCTGCCAACATTGAGGCTGTGGCAGCCAAGAACAAACACTGCCTGCT<br>GGAGGCTGGGATCGGCTGTGTGCGCGACCTGATCAAGTGCAAGGTGTACCCCATAGTGCTGCTCATCCGGGTGAGCGAGAAGAACATCAAACGGTTCAGGAAGCTGCTGCCGCGGCCAGAGACGGAAGAGGAATTCCTGCGAGTGTGCAGGCTCAAAGAGAAGGA<br>GCTGGAGGCGCTGCCCTGCCTCTACGCCACCGTGGAAGCTGAGATGTGGAGCAGCGTGGAGGAGCTGCTGCGAGTCCTCAAAGACAAGATTGTAGAGGAGCAGCGCAAGACCATCTGGGTGGACGAGGACCAGCTGTGA-3'			
HBZ		This paper	N/A
5'-<br>CAAAGACGCAGAGTCGAGCAAGCAGGGTCAGGCAAAGCGTGGAGAGCCGGCTGAGTCTAGGTAGGCTCCAAGGGAGCGCCGGACAAAGGCCCGGTCTCGACCTGAGCTTTAAACTTACCTAGACGGCGGACGCAGTTCAGGAGGCACCACAGGCGGGAGGCGGC<br>AGAACGCGACTCAACCGGCGTGGATGGCGGCCTCAGGGCTGTTTCGATGCTTGCCTGTGCCATGCCCGGAGGACCTGCTGGTGGAGGAATTGGTGGACGGGCTATTATCCTTGGAGGAAGAGTTAAAGGACAAGGAGGAGGAGGAAGCTGTGCTTGACGGTTTGC<br>TATCTTTAGAAGAGGAAAGCCGCGGCCGGCTGCGACGGGGCCCTCCAGGGGAGAAAGCGCCACCTCGCGGGGAAACGCATCGTGATCGGCAGCGACGGGCTGAGGAGAAGAGGAAGCGAAAAAAAGAGCGGGAGAAAGAGGAGGAAAAGCAGATTGCTGAGTAT<br>TTGAAAAGGAAGGAAGAGGAGAAGGCACGGCGCAGGAGGCGGGCGGAGAAGAAGGCCGCTGACGTCGCCCGGAGAAAGCAGGAAGAGCAGGAGCGCCGTGAGCGCAAGTGGAGACAAGGGGCTGAGAAGGCGAAACAGCATAGTGCTAGGAAAGAGAAAATGC<br>AGGAGTTGGGGATTGATGGTTATACTAGACAGTTGGAAGGCGAGGTGGAGTCCTTGGAGGCTGAACGGAGGAAGTTGCTGCAGGAGAAGGAGGATTTAATGGGAGAGGTTAATTATTGGCAGGGGAGGCTGGAGGCGATGTGGTTGCAA-3'			
Plasmids			
mRosa26 BAC vector (See Supplemental figure 1)		This paper	Geno Techs Co., #RP23-184A7; DDBJ, #LC739268
mCD4 promoter/enhancer vector (See Supplemental figure 1)		Dr. Iwakura (Tokyo University of Science)	DOI: 10.1006/viro.2001.0964.
Antibodies for Western blotting [dilution]			
Rabbit Anti-CARD11 (1D12)	[1:5000]	Cell Signaling Technology	#4435, RRID:AB_2070359
Rabbit Anti-Phospho-CARD11 (Ser652)	[1:5000]	Cell Signaling Technology	#5189, RRID:AB_10621241
Rabbit Anti-Cleaved BCL10	[1:5000]	Dr. D. Morishita (Chordia Therapeutics)	N/A
Rabbit Anti-Bcl10 (C78F1)	[1:5000]	Cell Signaling Technology	#4237, RRID:AB_2228005
Rabbit Anti-Phospho-NF-κB p65 (Ser536)	[1:5000]	Cell Signaling Technology	#3033, RRID:AB_331284
Rabbit Anti-RelA (C22B4)	[1:1000]	Cell Signaling Technology	#4764
Rabbit Anti-RelB (D7D7W)	[1:1000]	Cell Signaling Technology	#10544
Rabbit Anti-NF-κB1 p105/p50	[1:1000]	Cell Signaling Technology	#13586
Rabbit Anti-NF-κB2 p100/p52	[1:1000]	Cell Signaling Technology	#4882, RRID:AB_10695537
Rabbit Anti-IRF4 (D9P5H)	[1:1000]	Cell Signaling Technology	#15106
Rabbit Anti-HBZ	[1:1000]	Dr. Oshima (Tokushima Bunri University)	Muraki et al. Oncogene 33, 2317–2328 (2014).
Rabbit Anti-β-Actin (13E5) HRP conjugated	[1:1000]	Cell Signaling Technology	#5125, RRID:AB_1903890
Rabbit Anti-TBP	[1:1000]	Cell Signaling Technology	#44059
Rabbit Anti-α Tubulin	[1:1000]	Cell Signaling Technology	#2144
Goat Anti-Rabbit IgG HRP linked (secondary antibody)	[1:1000-5000]	Cell Signaling Technology	#7074, RRID:AB_2099233
Sheep Anti-Mouse IgG HRP linked (secondary antibody)	[1:1000-5000]	GE Healthcare	#NA9310V
Antibodies for flow cytometric analysis and cell sorting [dilution]			
Rat FITC Anti-Mouse/Human CD11b (Mac1)	[1:300]	BioLegend	#101206, RRID:AB_312789
Rat FITC Anti-Mouse CD41	[1:300]	BioLegend	#133903, RRID:AB_1626237
Rat FITC Anti-Mouse CD3	[1:300]	BioLegend	#100204, RRID:AB_312661
Rat FITC Anti-Mouse CD4	[1:300]	BioLegend	#100406, RRID:AB_312691
Rat PE Anti-Mouse Ly-6G/Ly-6C (Gr-1)	[1:600]	BioLegend	#108408, RRID:AB_313373
Rat PE Anti-Mouse Ter119	[1:600]	BioLegend	#116208, RRID:AB_313709
Rat PE Anti-Mouse CD4	[1:600]	BioLegend	#100408, RRID:AB_312693
Rat PE Anti-Mouse/Human CD45R/B220	[1:600]	BioLegend	#103208, RRID:AB_312993
Rat PE Anti-Mouse/Human CD44	[1:600]	BioLegend	#103008, RRID:AB_312959
Rat APC Anti-Mouse CD8a	[1:600]	BioLegend	#100712, RRID:AB_312751
Rat APC Anti-Mouse CD62L	[1:600]	BioLegend	#104412, RRID:AB_313099
Rat APC Anti-Mouse CD25	[1:600]	BioLegend	#102012, RRID:AB_312861
Rat Anti-Mouse CD16/CD32 (Mouse BD Fc Block)	[1:300]	BD Biosciences	#553141, RRID:AB_394656
7-AAD Viability Staining Solution	[1:100]	BioLegend	#420404
Antibodies for immunohistochemistry [dilution]			
Rabbit Anti-Mouse/Rat/Human CD3	[1:100]	Abcam	#ab16669, RRID:AB_443425
Rat Anti-Mouse CD45R (B220)	[1:100]	BD Biosciences	#550286, RRID:AB_393581
Rabbit Anti-CD44	[1:100]	proteintech	#15675-1-AP, RRID:AB_2076198
Rat Anti-Mouse/Rat FOXP3	[1:50]	eBioscience	#14-5773-82
Rabbit Anti-Mouse/Human Ki-67	[1:100]	Abcam	#ab15580, RRID:AB_443209
Goat Anti-Rabbit Immunoglobulins Biotinylated	[1:200]	Dako	#E0432, RRID:AB_2313609

Supplementary Table 2 (Continued). The reagents, genetically modified organisms and strains, cell lines, software, instrumentation, and source data.

REAGENT or RESOURCE	SOURCE	IDENTIFIER
Critical commercial assays		
Mouse CD4^{+} T Cell Isolation Kit	Miltenyi Biotec	#130-104-454
Nuclear Extract Kit	Active Motif	#40410
Nuclear/Cytosolic Fractionation Kit	Cell Biolabs	#AKR-172
Deposited Data		
Gene expression datasets obtained from mouse samples	This paper	DRA015050
Gene expression datasets obtained from human samples	Kataoka et al., 2015	EGAD00001001411
Software and Algorithms		
FlowJo v10.7.1	FlowJo, LLC	http://www.flowjo.com/
Kallisto v0.43.1	Bray et al., 2016	http://pachterlab.github.io/kallisto/
GSEA v4.1.0	Broad institute	https://software.broadinstitute.org/gsea/
MSigDB	Broad Institute	http://www.gsea-msigdb.org/gsea/msigdb/index.jsp
Signature DB	NIH	https://lymphochip.nih.gov/signaturedb/
R version 4.0.3	R Core Team	www.r-project.org
edgeR v3.12	Robinson et al., 2010	https://bioconductor.org/
Enrichr	Chen et al., 2013	https://maayanlab.cloud/Enrichr/
iDEP v0.92	Ge et al., 2018	http://bioinformatics.sdstate.edu/idep/
Marbach2016	Marbach et al., 2016	https://rdrr.io/github/slowkow/tftargets/man/Marbach2016.html
TRRUST v2	Han et al., 2018	https://www.grnpedia.org/trrust/
ComBat-seq	Y Zhang et al., 2020	https://github.com/zhangyuqing/ComBat-seq

Supplementary Figure 1

(a)

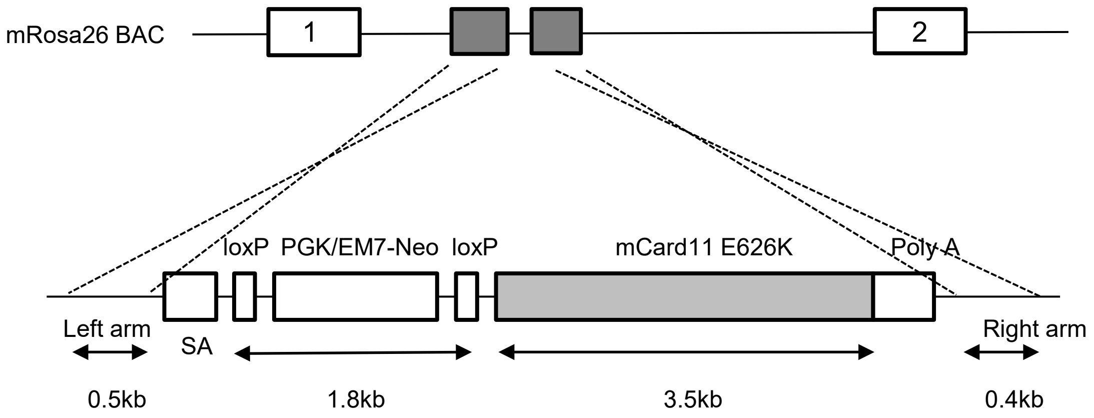

(b)

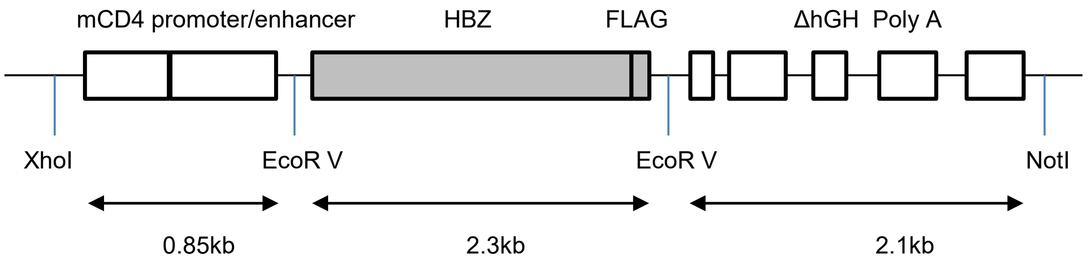

Supplementary Figure legends

Supplementary Figure 1. Strategy for targeting mutant mice.

(a) Strategy for targeting CARD11(E626K)$^{\mathrm{stop}FL}$ mice. *Card11*E626K cDNA, which is a murine homologue of human *CARD11*E616K, was inserted into the *mRosa26* vector, preceded by a loxP-flanked (FL) stop sequence. CARD11(E626K)$^{CD4\text{-}\mathrm{Cre}}$ mice were obtained by crossing CARD11(E626K)$^{\mathrm{stop}FL}$ mice with *CD4*-Cre transgenic (Tg) mice. SA, splice acceptor; loxP, loxP sequences; PGK, phosphoglycerate kinase eukaryotic promoter; EM7, EM7 prokaryotic promoter; Neo, neomycin resistance gene.

(b) Strategy for targeting *HBZ* Tg mice. The original transgene construct containing a mouse *CD4* promoter/enhancer, *vpr* (an accessory protein of HIV), and a defective human growth hormone gene with the polyadenylation signal were kindly provided by Dr. Iwakura from Tokyo University of Science(Yasuda et al., 2001). The *vpr* of the transgene construct was replaced by *HBZ-FLAG* after digestion with EcoRV and SmalI. The *HBZ* transgene construct was prepared for microinjection after digestion with XhoI and NotI.

Supplementary Figure 2 (a)

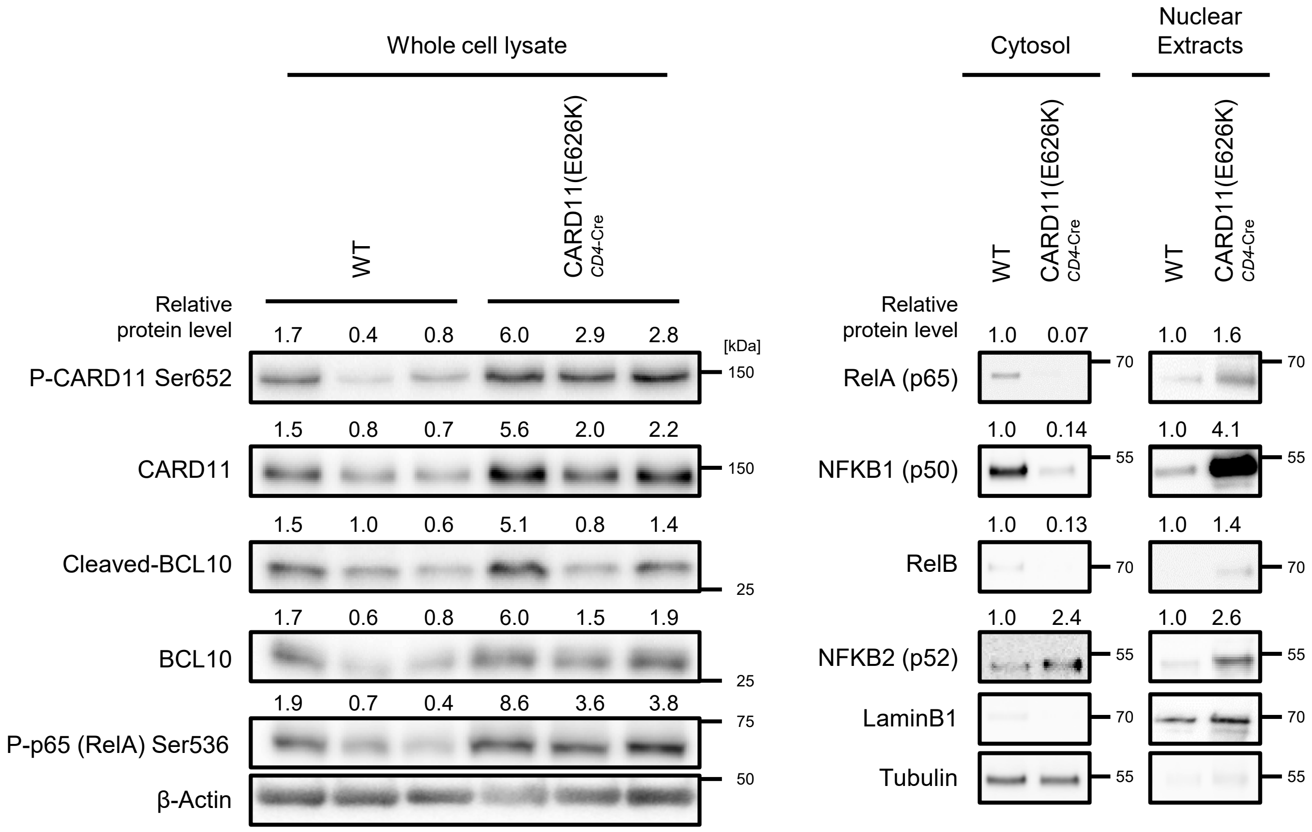

(b)

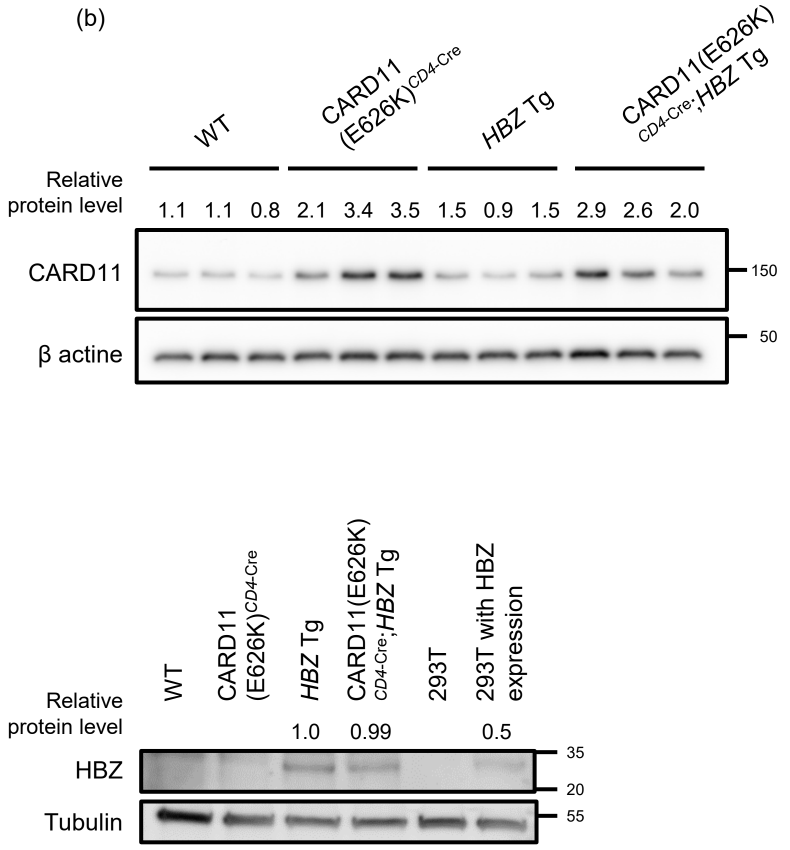

Supplementary Figure 2. Western blot analysis of splenic CD4$^+$ T cells.

(a) Activation of the NF-κB pathway in CARD11(E626K)$^{\mathit{CD4}\text{-Cre}}$ mice. (left) Immunoblots of MACS-purified splenic CD4$^+$ T cells from CARD11(E626K)$^{\mathit{CD4}\text{-Cre}}$ mice. Phosphorylated CARD11, the cleaved form of BCL10, and phosphorylated RelA (p65) were assessed. (right) Immunoblots of cytoplasmic and nuclear fractions in MACS-purified splenic CD4$^+$ T cells from CARD11(E626K)$^{\mathit{CD4}\text{-Cre}}$ mice. Nuclear translocation of NF-κB proteins (RelA, p50, RelB, and p52) were assessed. The expression level of each protein was normalized to β-actinin in the whole cell lysate, to tubulin in the cytosolic fraction, and to lamin-B1 in the nuclear fraction, and the relative ratios of the indicated proteins in CARD11(E626K)$^{\mathit{CD4}\text{-Cre}}$ mice to WT mice are shown.

(b) Expression levels of CARD11 and HBZ in splenic CD4$^+$ T cells from WT, CARD11(E626K)$^{\mathit{CD4}\text{-Cre}}$, *HBZ* Tg, and CARD11(E626K)$^{\mathit{CD4}\text{-Cre}}$;*HBZ* Tg mice. Immunoblots for CARD11 (upper) and HBZ (lower) in MACS-purified splenic CD4$^+$ T cells from 4 mouse types. Protein expression levels were normalized to ß-actin, and relative ratios of CARD11 in each mouse type to WT mice are shown. Cell lysates of 293T cells expressing HBZ were used as positive controls for HBZ western blotting.

HBZ expression levels were normalized to tubulin, and relative ratios of HBZ in CARD11(E626K)$^{CD4\text{-Cre}}$;*HBZ* Tg mice or 293T cells expressing HBZ to *HBZ* Tg mice are shown.

Supplementary Figure 3

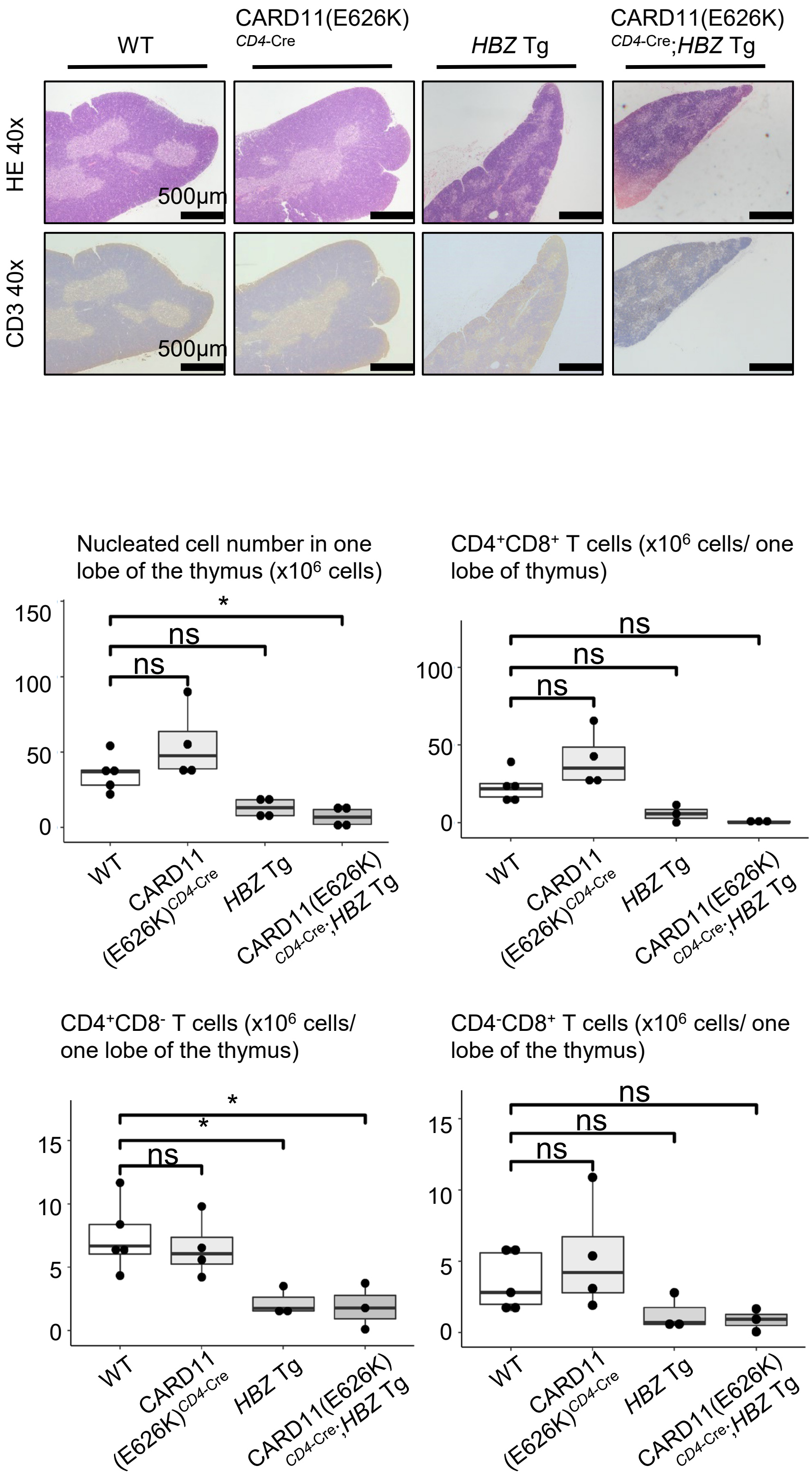

Supplementary Figure 3. Thymic atrophy in CARD11(E626K)$^{\mathit{CD4}\text{-Cre}}$;*HBZ* Tg mice.

Pathology, cellularity, and T-cell number in the thymus in WT (n = 5), CARD11(E626K)$^{\mathit{CD4}\text{-Cre}}$ (n = 4), *HBZ* Tg (n = 4), and CARD11(E626K)$^{\mathit{CD4}\text{-Cre}}$;*HBZ* Tg mice (n = 4) at 6 months after birth. Thymic sections were stained with HE or anti-CD3 antibodies, and the number of nucleated cells in one lobe of the thymus was counted. Absolute numbers of $CD4^+CD8^+$, $CD4^+CD8^-$, and $CD4^-CD8^+$ thymic T cells are shown. p values were calculated by Tukey's test after one-way ANOVA; * represent p values less than 0.05.

Supplementary Figure 4

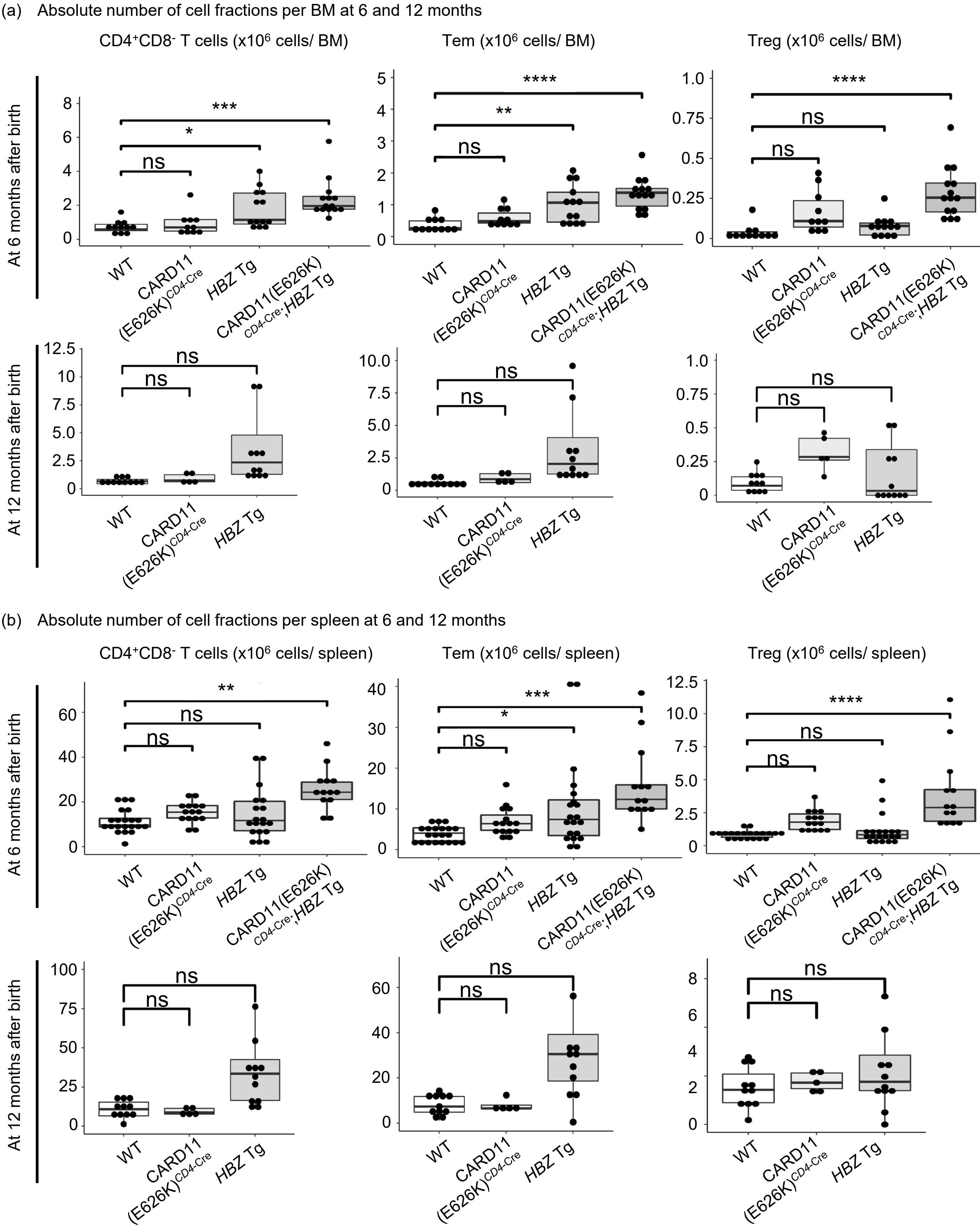

Supplementary Figure 4 (continued)

(c) Absolute number of cell fractions per BM at 6 and 12 months after birth

CD4$^-$CD8$^+$ T cells (x10^6 cells/ BM)

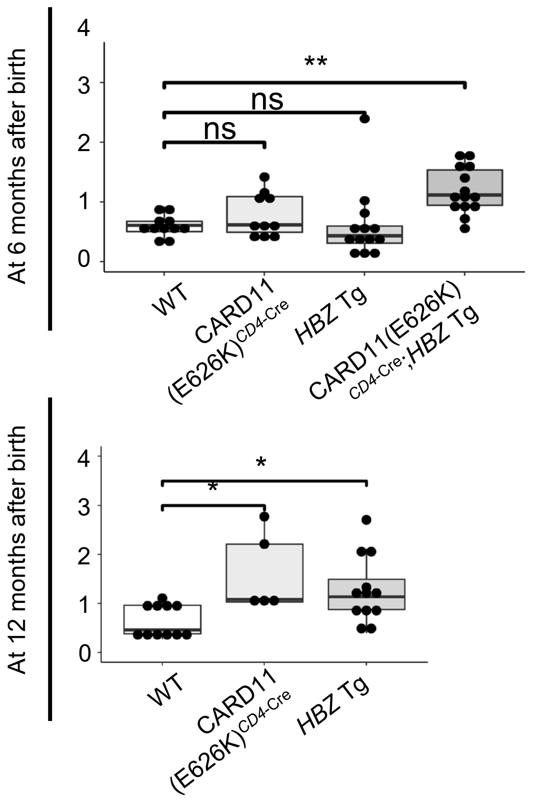

(d) Absolute number of cell fractions per spleen at 6 and 12 months after birth

CD4$^-$CD8$^+$ T cells (x10^6 cells/ spleen)

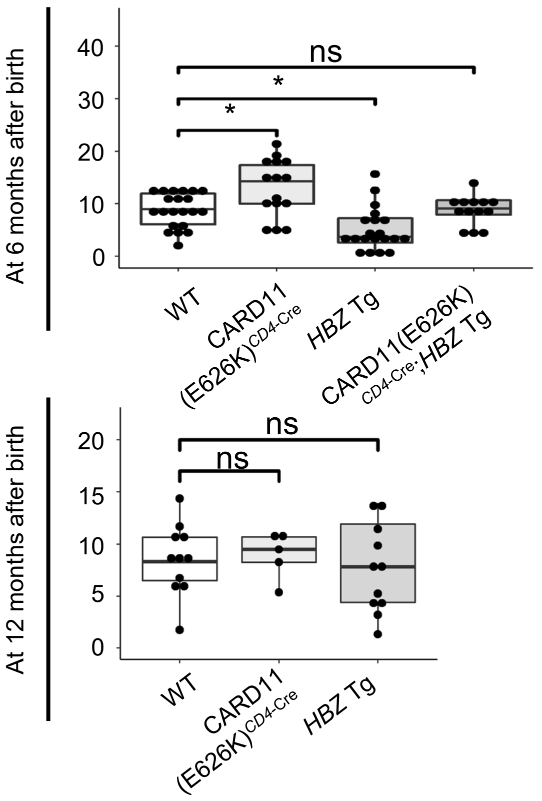

Supplementary Figure 4. T-cell subpopulation analysis in BM and spleen.

Absolute numbers of CD4$^+$CD8$^-$ T cells, CD4$^+$CD44$^+$CD62L$^-$ Tem, and CD4$^+$CD25$^+$ Treg (a, b), and CD4$^-$CD8$^+$ T cells (c, d), in BM (a, c) and spleen (b, d) from WT (n = 11), CARD11(E626K)$^{CD4\text{-}Cre}$ (n = 10) *HBZ* Tg (n = 13), and CARD11(E626K)$^{CD4\text{-}Cre}$;*HBZ* Tg (n = 14) mice at 6 months after birth, and from WT (n = 11), CARD11(E626K)$^{CD4\text{-}Cre}$ (n = 5), and *HBZ* Tg (n = 12) mice at 12 months after birth. p values were calculated by the Tukey test after a one-way ANOVA; *, **, *** represent p values less than 0.05, 0.01, and 0.001, respectively.

Supplementary Figure 5

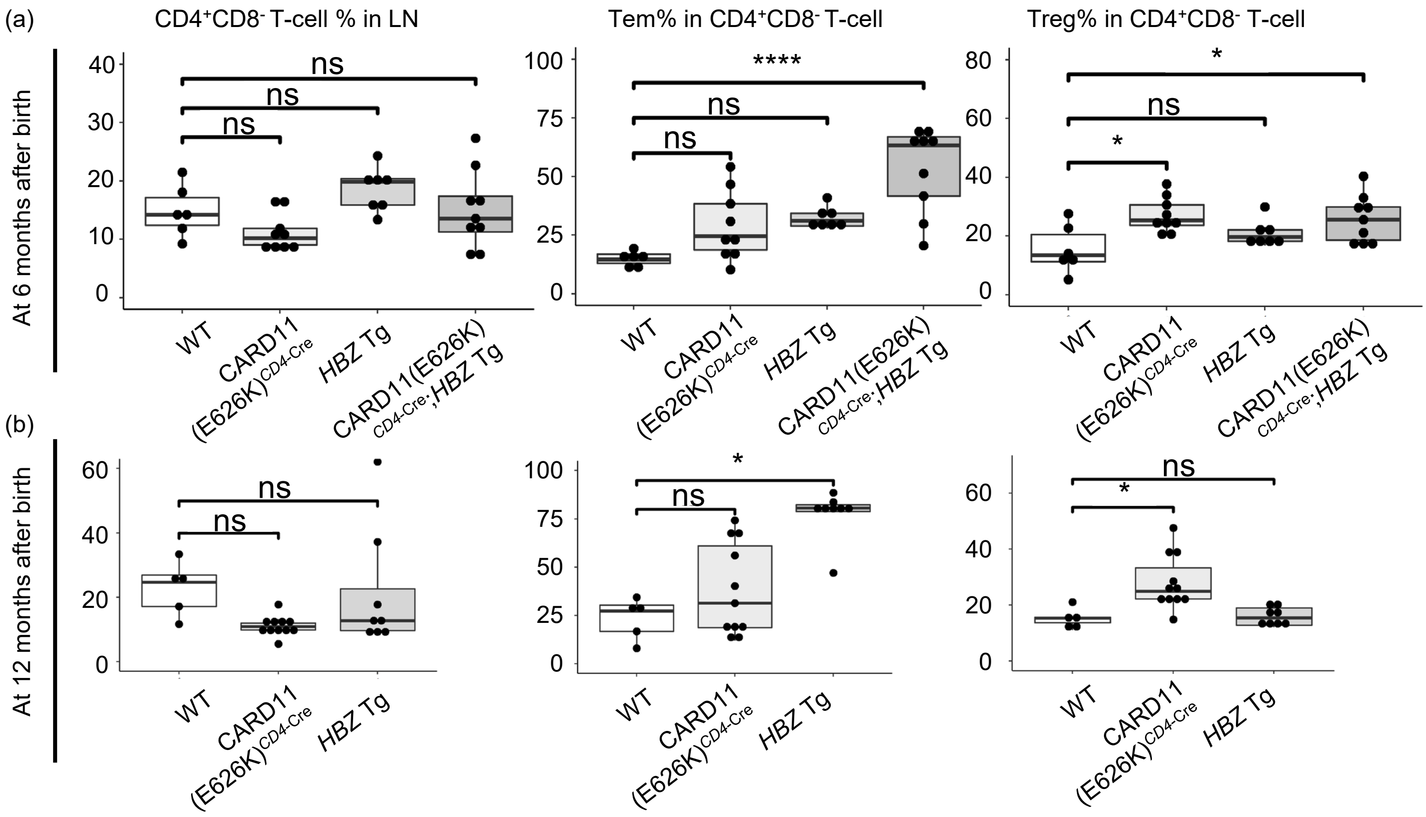

Supplementary Figure 5. Abnormal increments of the relative percentages of effector/memory T cells and regulatory T cells in lymph nodes of CD4$^+$ T cells in mutant mice.

The percentage of CD4$^+$CD8$^-$ T cells relative to the total number of nucleated cells in lymph node (LN), and the relative percentages of CD4$^+$CD44$^+$CD62L$^-$ effector/memory T cells (Tem%) and CD4$^+$CD25$^+$ regulatory T cells (Treg%) in the CD4$^+$CD8$^-$ T-cell subset. These percentages are shown at (a) 6 months after birth (wild type (WT), n = 6; CARD11(E626K)$^{CD4\text{-Cre}}$,n = 9; *HBZ* Tg, n = 7; and CARD11(E626K)$^{CD4\text{-Cre}}$;*HBZ* Tg, n = 9 mice) and at (b) 12 months after birth (WT, n = 5; CARD11(E626K)$^{CD4\text{-Cre}}$,n = 11; *HBZ* Tg, n = 8). p values were calculated by the Tukey test after a one-way ANOVA; *, **, *** represent p values less than 0.05, 0.01, and 0.001, respectively.

Supplementary Figure 6

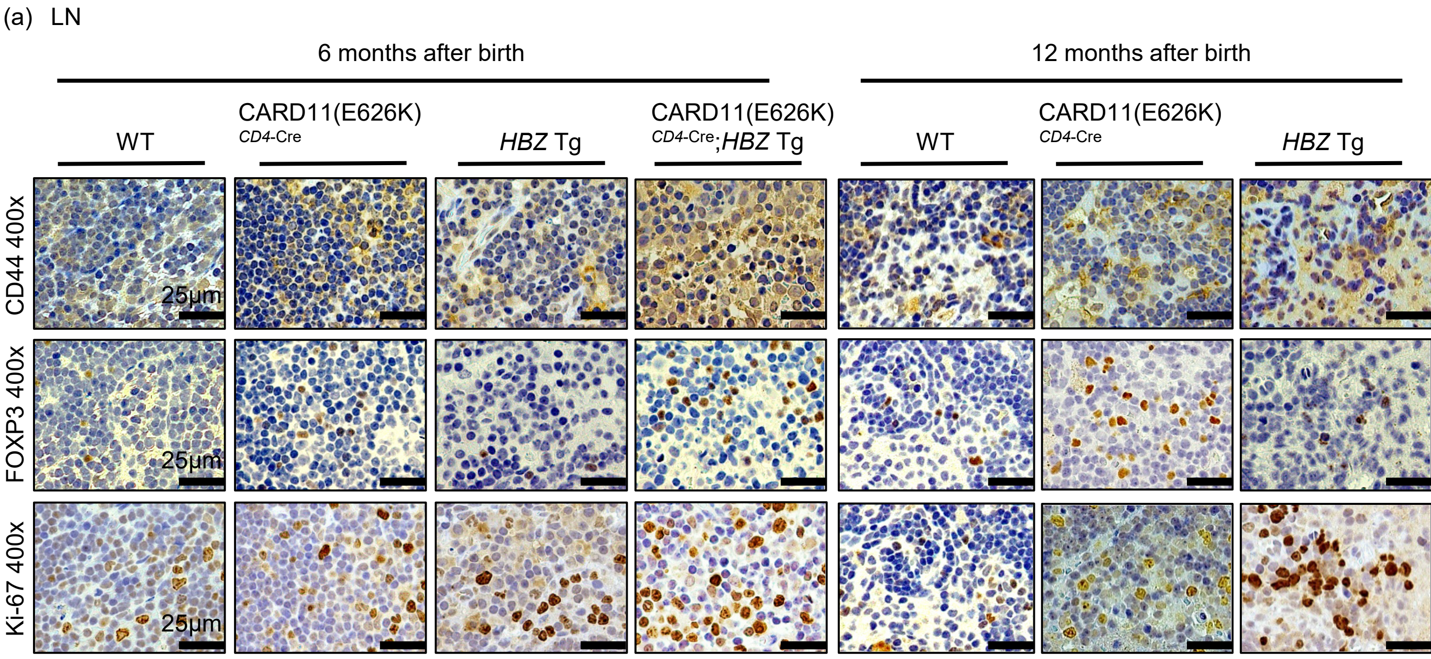

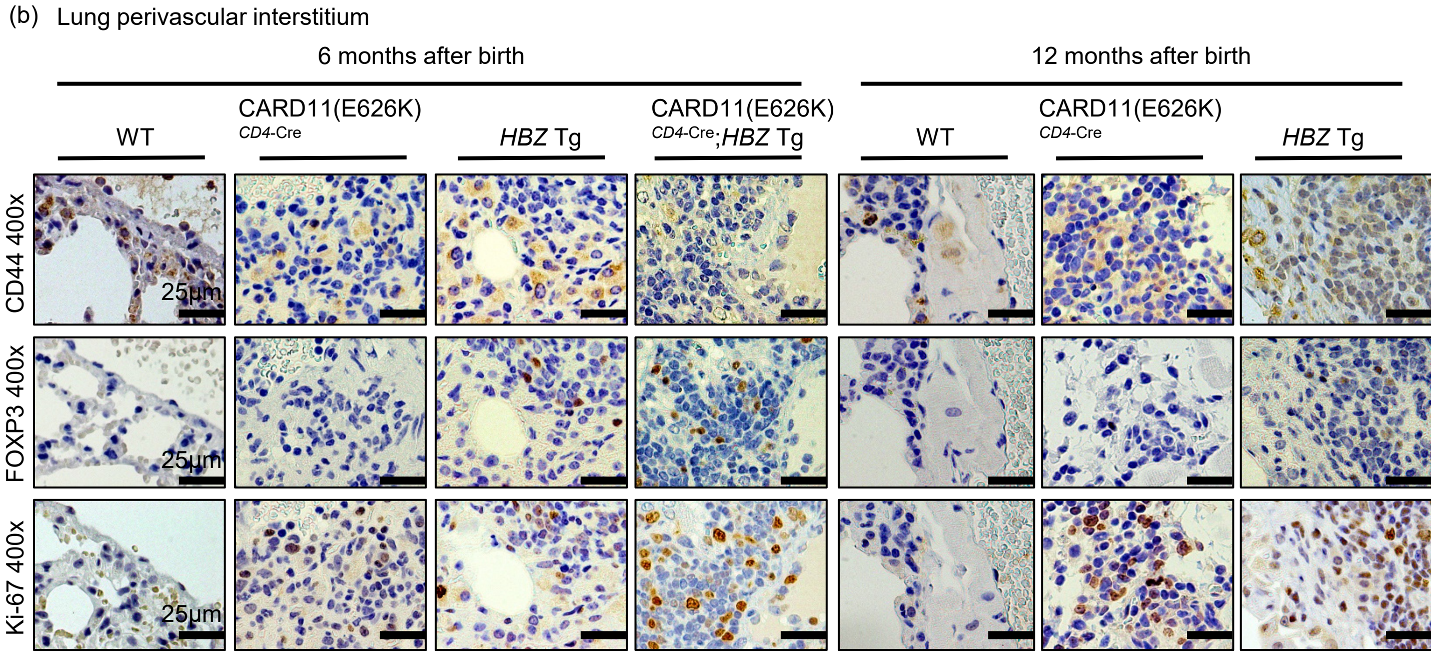

Supplementary Figure 6 (continued)

(c)

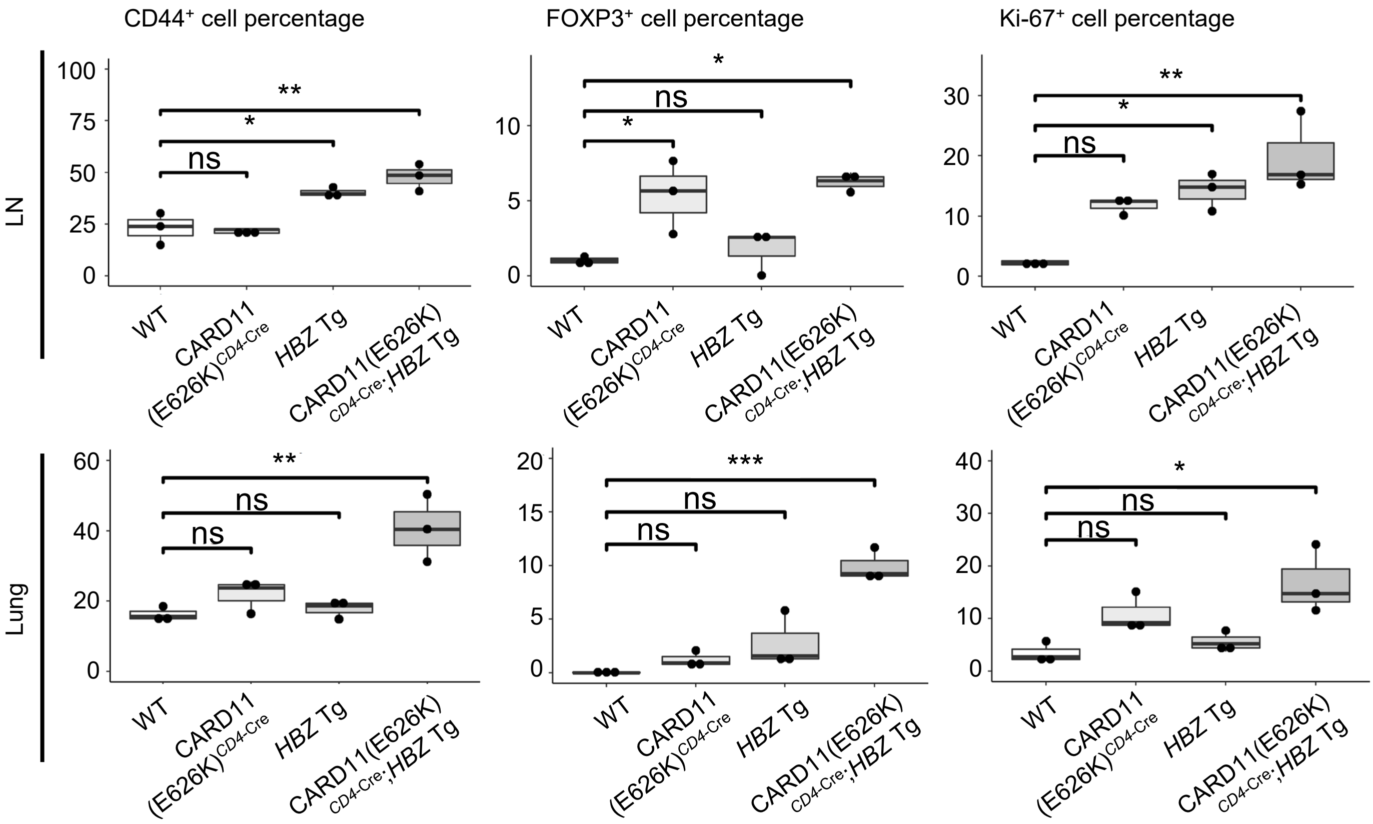

(d)

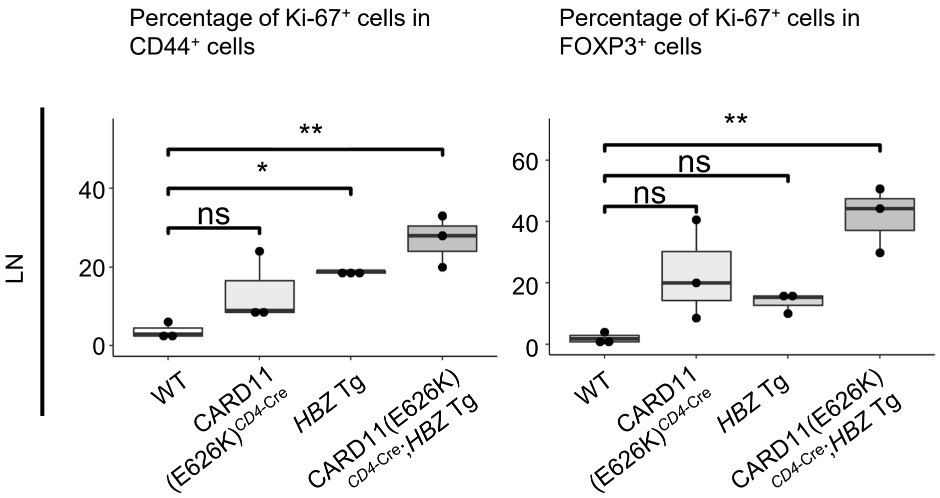

Supplementary Figure 6. Increase of both Tem and Treg in LNs and lungs in CARD11(E626K)$^{\mathit{CD4}\text{-Cre}}$;*HBZ* Tg mice.

(a, b) Consecutive sections of LNs and lung perivascular interstitium stained by anti-CD44, anti-FOXP3, or anti-Ki-67 antibody at 6 or 12 months after birth. (c) Quantitative assessment of the frequencies of CD44^{+}, FOXP3^{+}, or Ki-67^{+} cells in LNs and lung perivascular interstitium at 6 months after birth. The percentage of CD44^{+}, FOXP3^{+}, or Ki-67^{+} cells relative to the total number of nucleated cells was scored in 5 microscopic views per slide from 3 mice of each type. (d) Ki-67 positivity in CD44+ cells or FOXP3+ cells in LNs at 6 months after birth. Ki-67 positivity were quantitatively scored in 100 CD44+ cells or FOXP3+ cells per slide from 3 mice of each type. p values were calculated by the Tukey's test after one-way ANOVA; *, **, *** represent p values less than 0.05, 0.01, and 0.001, respectively.

Supplementary Figure 7

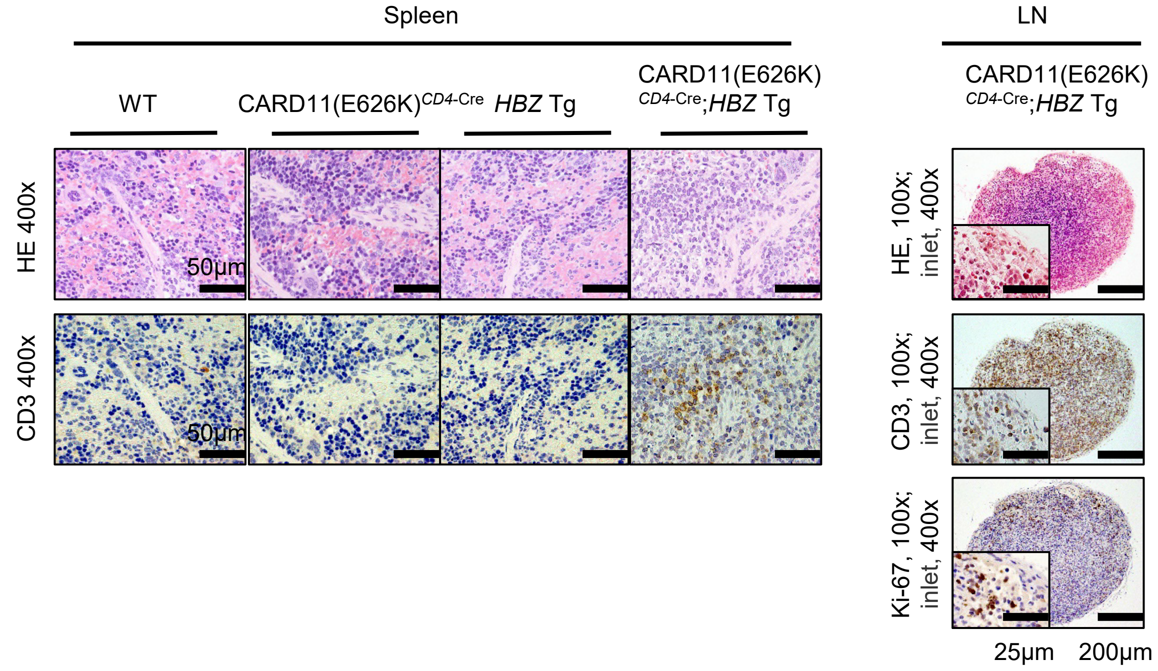

Supplementary Figure 7. T-cell growth in recipient mice transplanted with $CD4^{+}$ cells from CARD11(E626K)$^{CD4\text{-}Cre}$;*HBZ* Tg mice.

Pathological assessment of the spleen and LNs in recipient mice transplanted with $CD4^{+}$ T cells of each mouse type. MACS-purified splenic $CD4^{+}$ T cells from each mouse type were transplanted to NOG mice. At 18 weeks after transplantation, overt tumors were not visible in recipient mice, but small LNs were detected in recipient mice transplanted with CARD11(E626K)$^{CD4\text{-}Cre}$;*HBZ* Tg $CD4^{+}$ cells. Spleen samples were stained by HE and anti-CD3 antibodies. Representative findings of a perivascular region are shown (400x). LNs were stained by HE, anti-CD3 antibodies, and anti-Ki-67 antibodies (100x; inlet, 400x).

Supplementary Figure 8

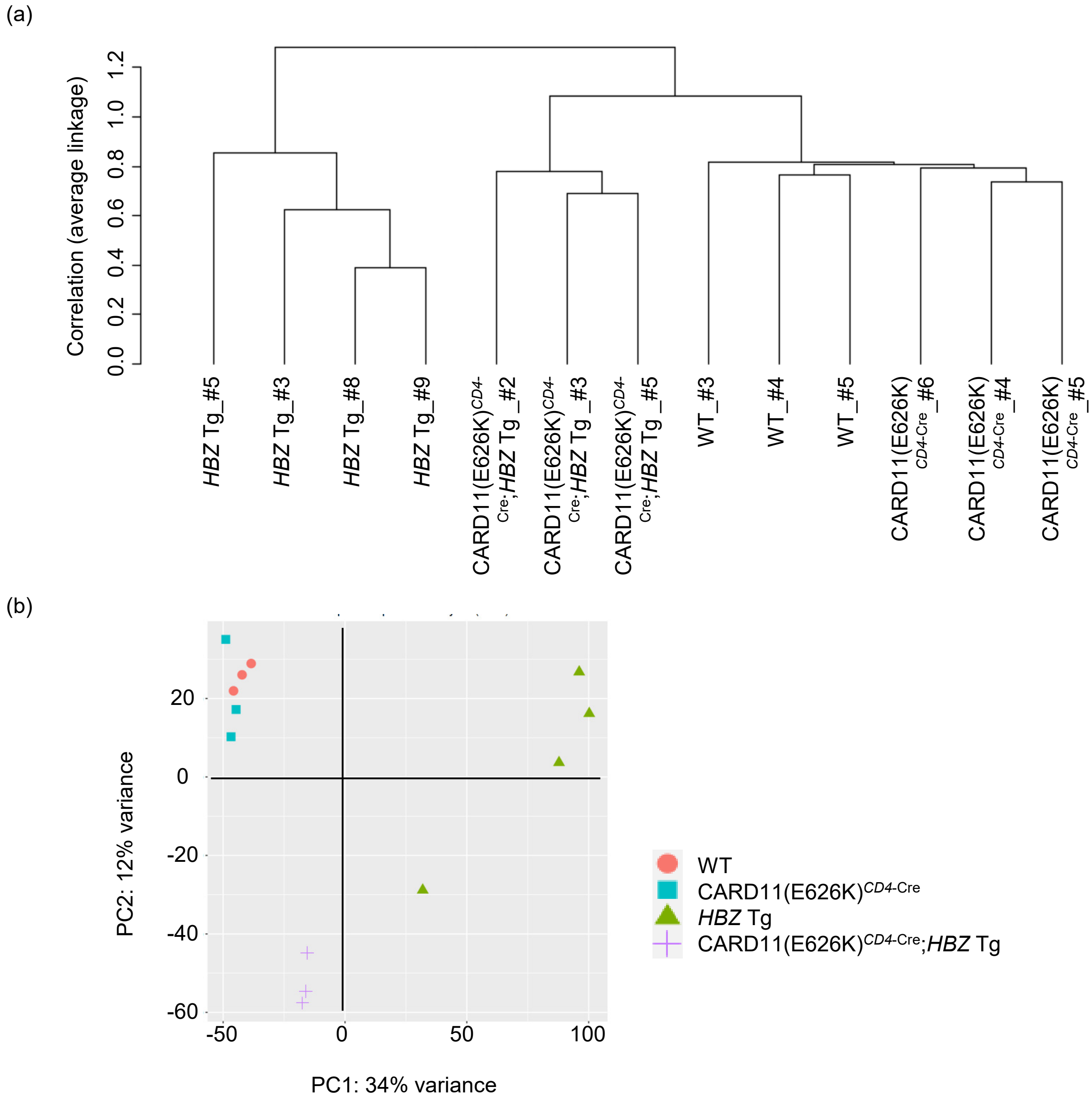

Supplementary Figure 8. Global gene expression profiling of Tem.

(a) Dendrogram constructed from unsupervised hierarchical clustering of RNA-seq data from 4 types of Tem using Pearson correlation. (b) Principal component analysis of RNA-seq data from 4 types of Tem. Samples were obtained from WT (n = 3), CARD11(E626K)$^{CD4\text{-}Cre}$ (n = 3), *HBZ* Tg (n = 4), and CARD11(E626K)$^{CD4\text{-}Cre}$;*HBZ* Tg (n = 3) mice at 4–6 months after birth.

Supplementary Figure 9

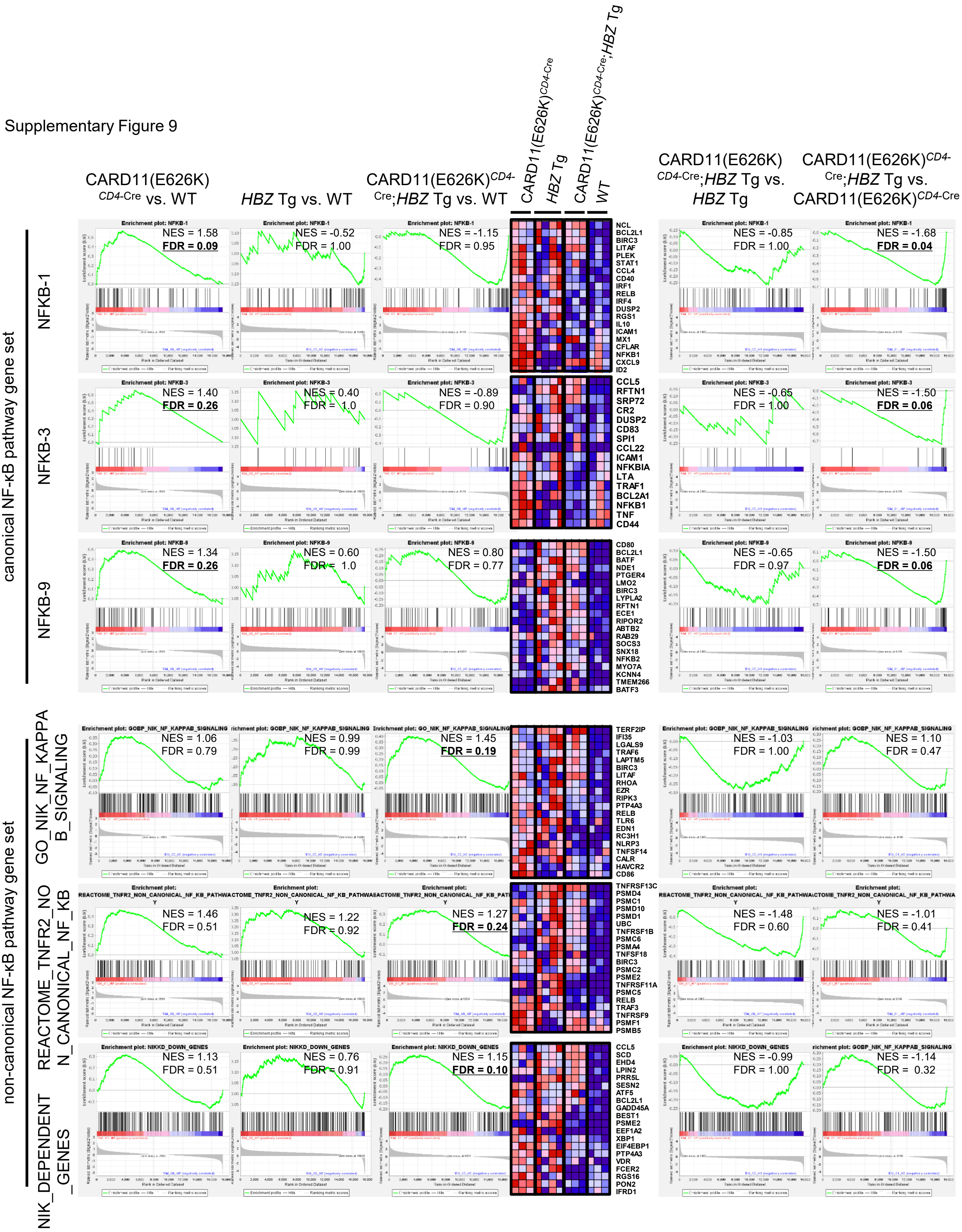

Supplementary Figure 9. Activation of the NF-κB canonical signaling pathway in CARD11(E626K)$^{\mathit{CD4}\text{-Cre}}$ mice, and that of the NF-κB non-canonical signaling pathway in CARD11(E626K)$^{\mathit{CD4}\text{-Cre}}$;*HBZ* Tg mice.

Gene set enrichment analysis (GSEA) of splenic $CD4^{+}CD44^{+}CD62L^{-}$ effector/memory T cells (Tem) from CARD11(E626K)$^{\mathit{CD4}\text{-Cre}}$, *HBZ* Tg, and CARD11(E626K)$^{\mathit{CD4}\text{-Cre}}$;*HBZ* Tg mice, compared with those from wild-type (WT) mice. Enrichment plots of gene sets related to the canonical NF-κB pathway (NFKB-1, NFKB-3, and NFKB-9 gene sets (SignatureDB)) and the non-canonical NF-κB pathway (GO_NIK_NF_KAPPAB_SIGNALING gene set, REACTOME_TNFR2_NON_CANONICAL_NF_KB_PATHWAY, and NIK_DEPENDENT_GENES) are shown with normalized enrichment scores (NESs) and FDRs[6,7,10]. GSEA was performed using a FDR cut-off of ≤0.25. Heat map shows the 20 most differentially expressed genes between CARD11(E626K)$^{\mathit{CD4}\text{-Cre}}$;*HBZ* Tg mice and WT mice in the gene set ranked by GSEA.

Supplementary Figure 10

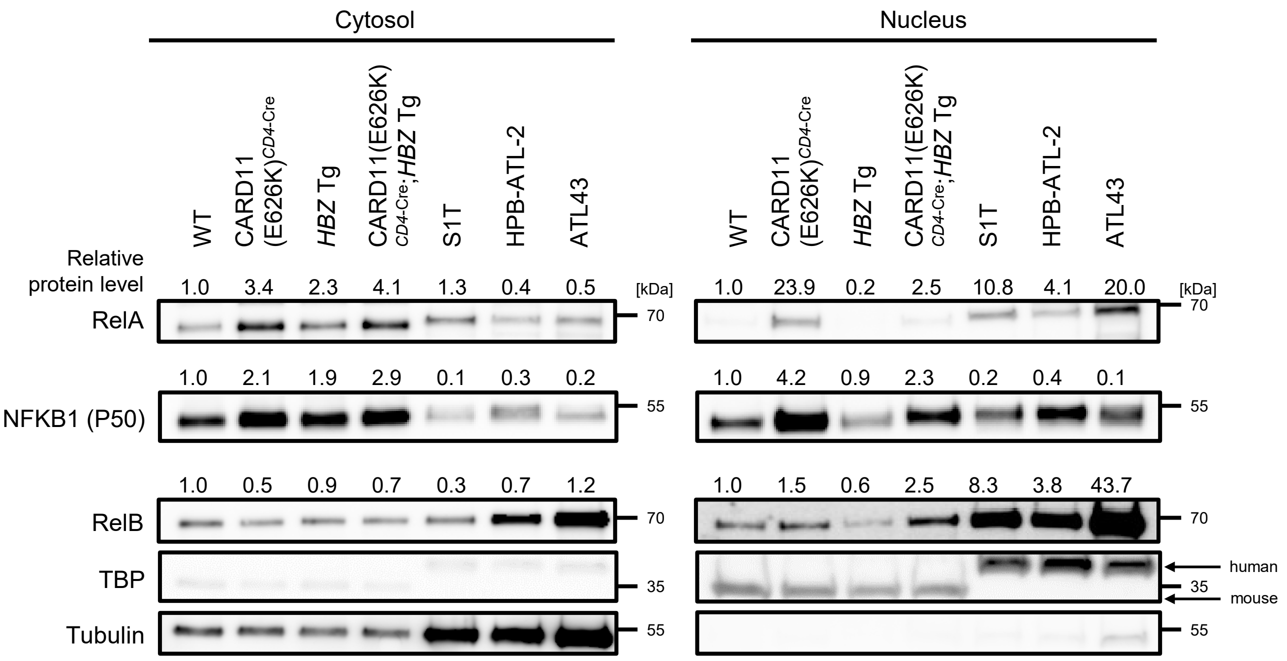

Supplementary Figure 10. Western blotting for NF κ B pathway proteins.

Immunoblots of canonical NF-κB pathway proteins RelA and NFKB1 (p50), and non-canonical pathway protein RelB in cytoplasmic and nuclear extractions from MACS-purified splenic CD4+ T cells of WT, CARD11(E626K)*CD4*-Cre, *HBZ* Tg, and CARD11(E626K)*CD4*-Cre;*HBZ* Tg mice. Protein expression levels were normalized to tubulin for cytoplasmic proteins, and to TATA-binding protein (TBP) for nuclear proteins. The relative ratio of each NF-κB protein in mutant mice to WT mice is shown. Cytoplasmic or nuclear extractions of ATL cell lines (S1T, HPB-ATL-2, ATL43) are presented as positive controls.

Supplementary Figure 11

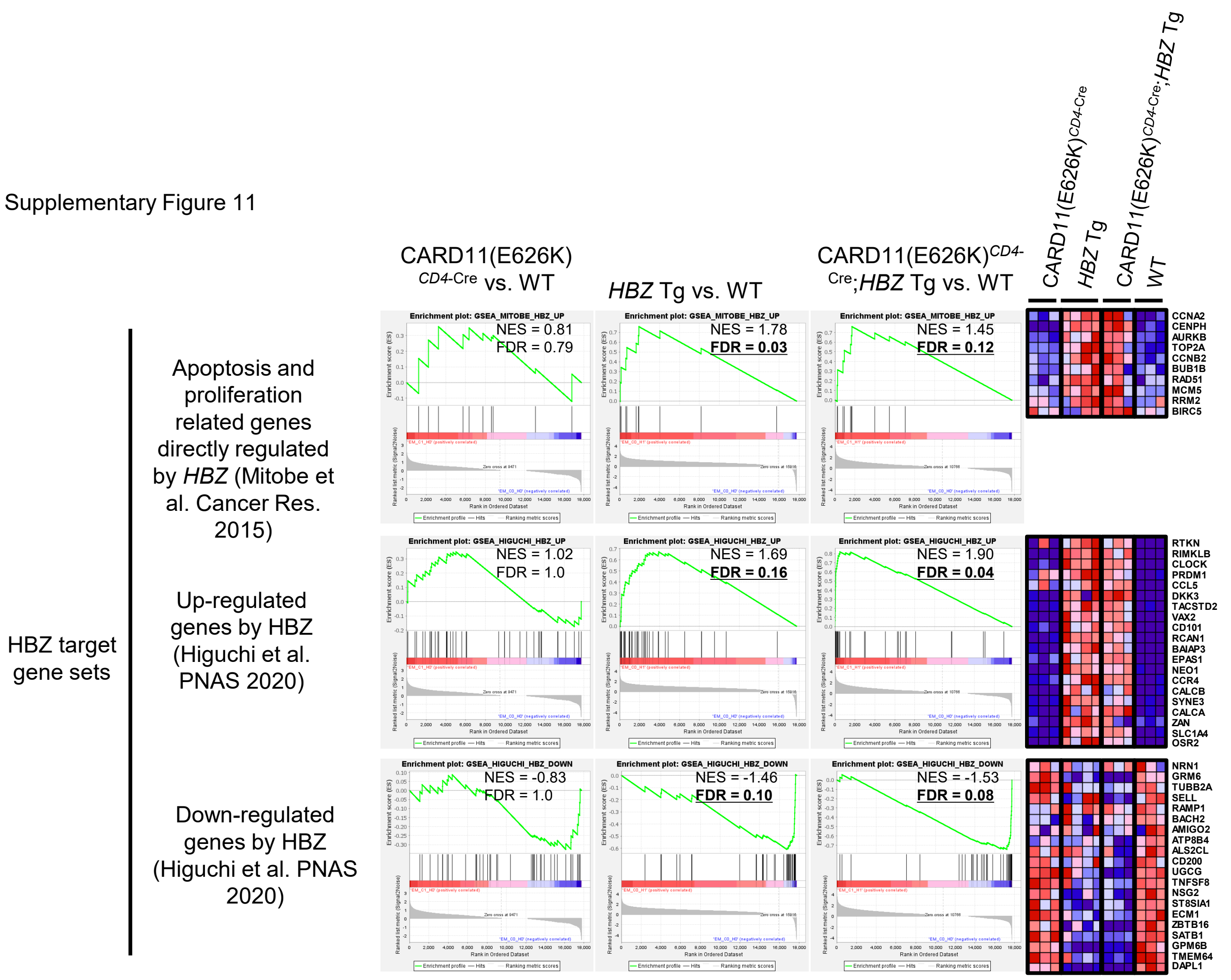

Supplementary Figure 11. Dysregulation of HBZ target genes in *HBZ* Tg and CARD11(E626K)*CD4*-Cre;*HBZ* Tg mice.

Characteristic apoptosis- and proliferation-related genes that are directly upregulated by *HBZ mRNA*[11] are enriched in both *HBZ* Tg mice and CARD11(E626K)*CD4*-Cre;*HBZ* Tg mice. Genes up- or downregulated by HBZ[12], including indirectly, are enriched in both *HBZ-Tg* and CARD11(E626K)*CD4*-Cre;*HBZ* Tg mice.

Supplementary Figure 12

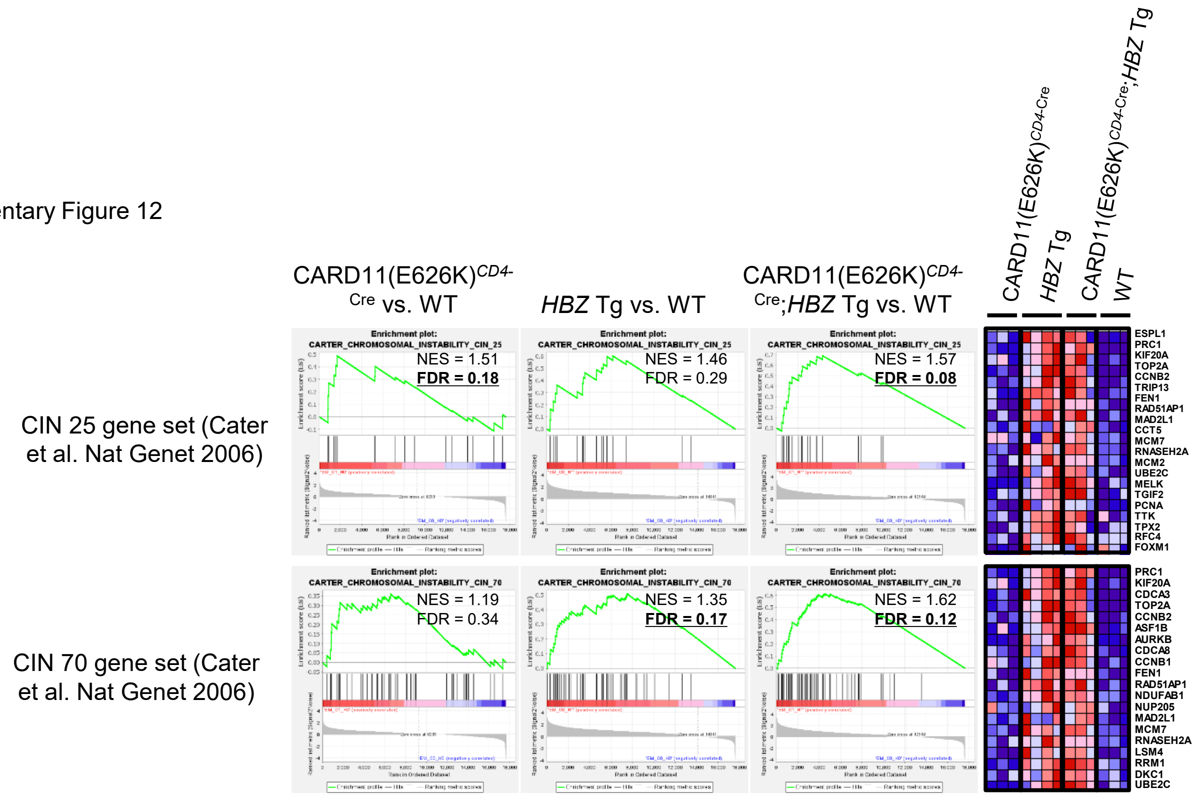

Supplementary Figure 12. Enrichment of genomic instability-related genes in CARD11(E626K)*CD4-*Cre;*HBZ* Tg mice

GSEA analysis of samples from each mouse type, with gene sets consisting of the top 25 and top 70 genes whose expression levels correlate with chromosomal instability in cancers[13].

Supplementary Figure 13

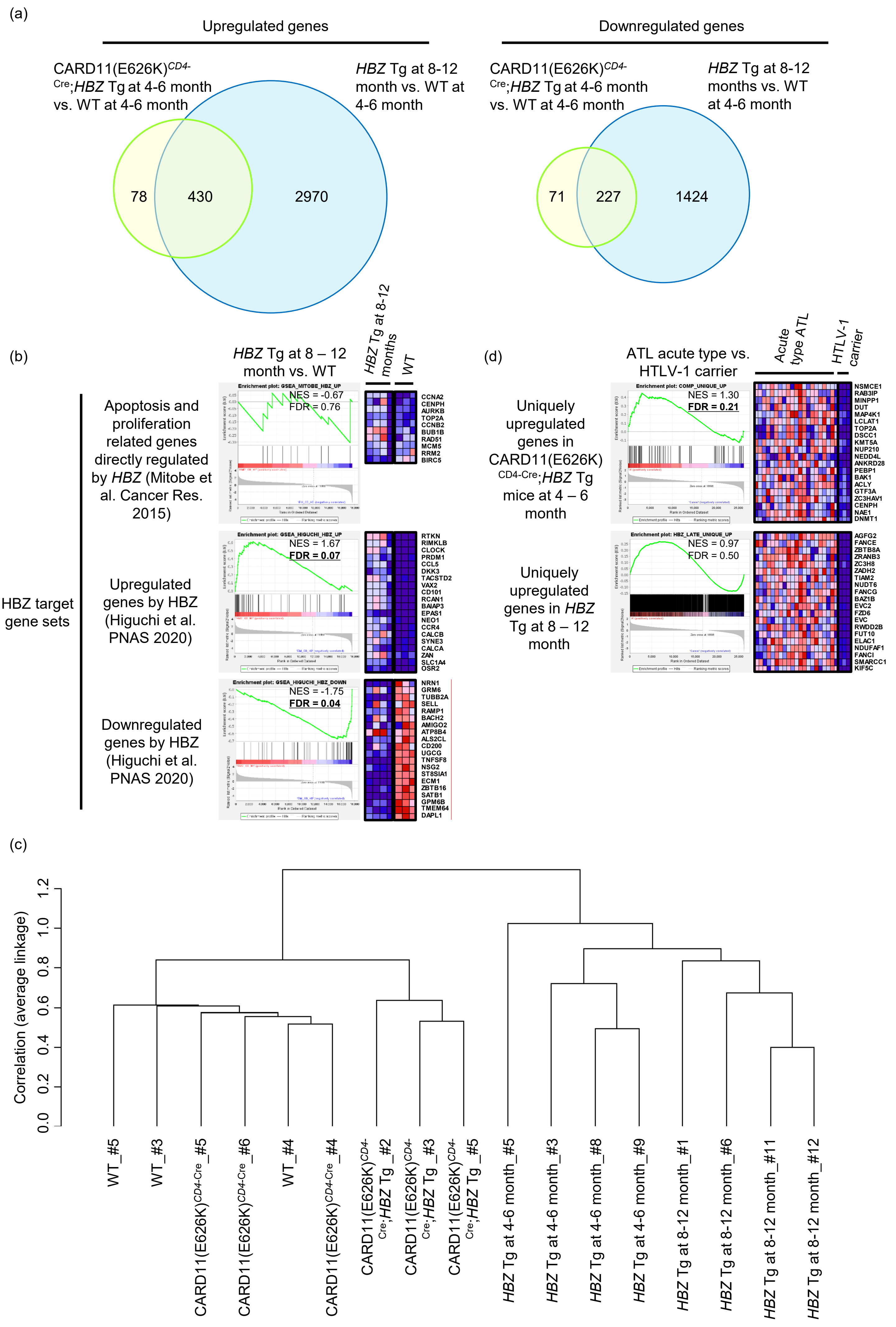

Supplementary Figure 13. Comparison of mRNA expression profiles in Tem between CARD11(E626K)$^{\mathit{CD4}\text{-Cre}}$;*HBZ* Tg mice at 4–6 months and *HBZ* Tg mice at 8–12 months.

(a) Venn diagrams of the overlap between significantly up- and downregulated genes in CARD11(E626K)$^{\mathit{CD4}\text{-Cre}}$;*HBZ* Tg mice at 4–6 months (n=3) vs. WT mice at 4–6 months (n=3), and *HBZ*-Tg mice at 8–12 months (n=4) vs. WT mice at 4–6 months (n=3). Splenic Tem were sorted and underwent expression analysis. Differentially expressed genes between mouse types were identified using cut-offs of fold change >1.2 and FDR <0.1.

(b) Dysregulation of HBZ target genes in *HBZ* Tg mice at 8–12 months (see also Supplementary Figure 11)[11,12].

(c) Hierarchical clustering analysis of mouse types, including *HBZ*-Tg mice at 8–12 months (see also Supplementary Figure 8). *HBZ* Tg at 8–12 months comprised a different gene expression hierarchy branch than CARD11(E626K)$^{\mathit{CD4}\text{-Cre}}$;*HBZ* Tg mice at 4–6 months.

(d) GSEA analysis of human acute-type ATL samples, using gene sets whose expression was uniquely activated in CARD11(E626K)$^{\mathit{CD4}\text{-Cre}}$;*HBZ* Tg mice at 4–6 months or *HBZ* Tg mice at 8–12 months. Uniquely upregulated gene sets in CARD11(E626K)$^{\mathit{CD4}\text{-Cre}}$;*HBZ* Tg mice at 4–6 months (n = 78) were enriched in acute-type ATL patient

samples compared with HTLV-1 carrier samples, while uniquely upregulated gene sets in *HBZ* Tg at 8–12 months (n = 2970) did not exhibit this enrichment.

Supplementary Figure 14

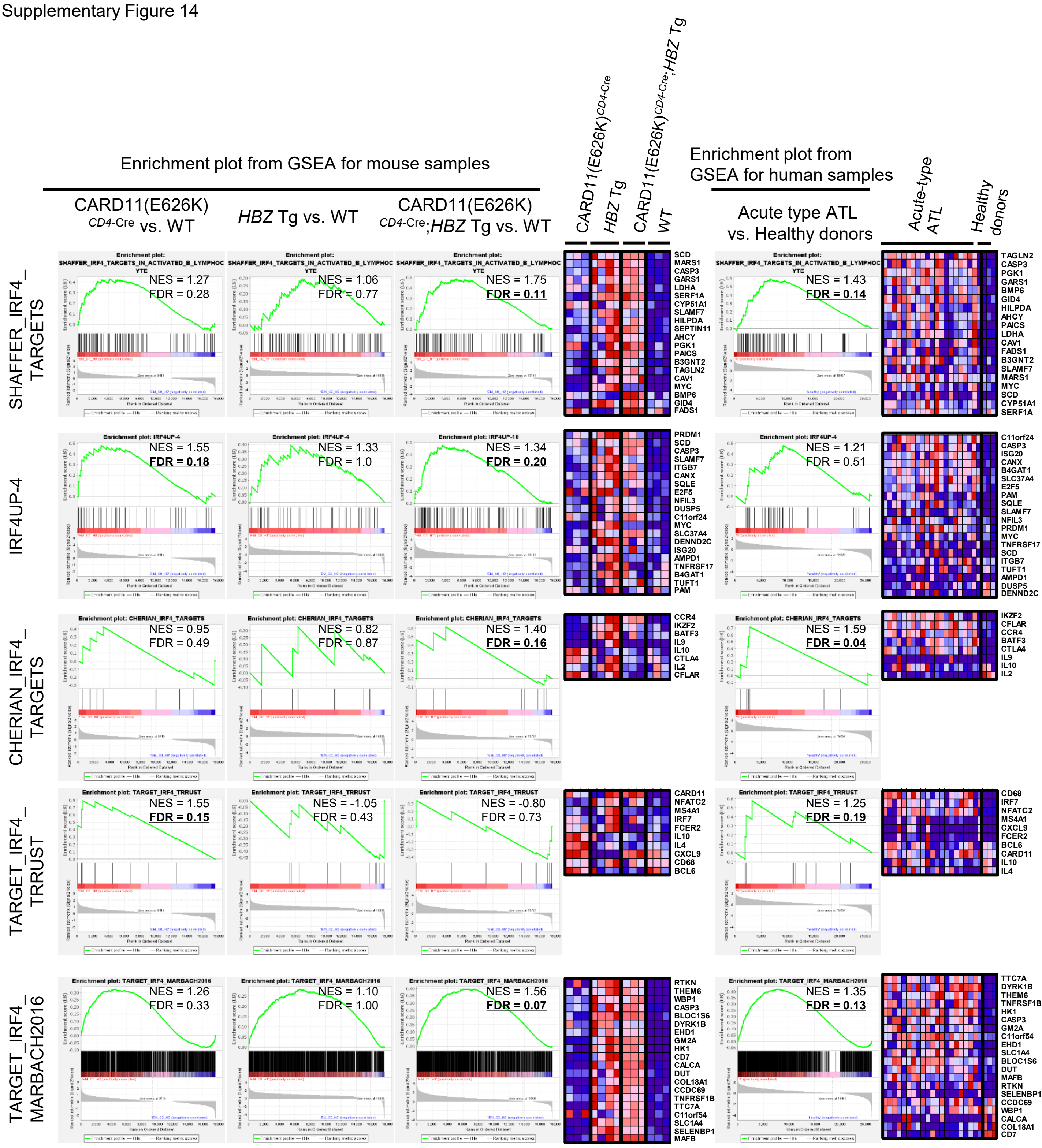

Supplementary Figure 14. Enrichment of IRF4 target gene sets in CARD11(E626K)*CD4-Cre*;*HBZ* Tg mice and human acute-type ATL patient samples.

GSEA analysis of IRF4 target genes in mutant mice and human acute-type ATL samples. IRF4 target gene sets were collected from the following databases: msigDB, signitureDB, TRRUST, and Marbach2016[6,7,14,15].

Supplementary Figure 15

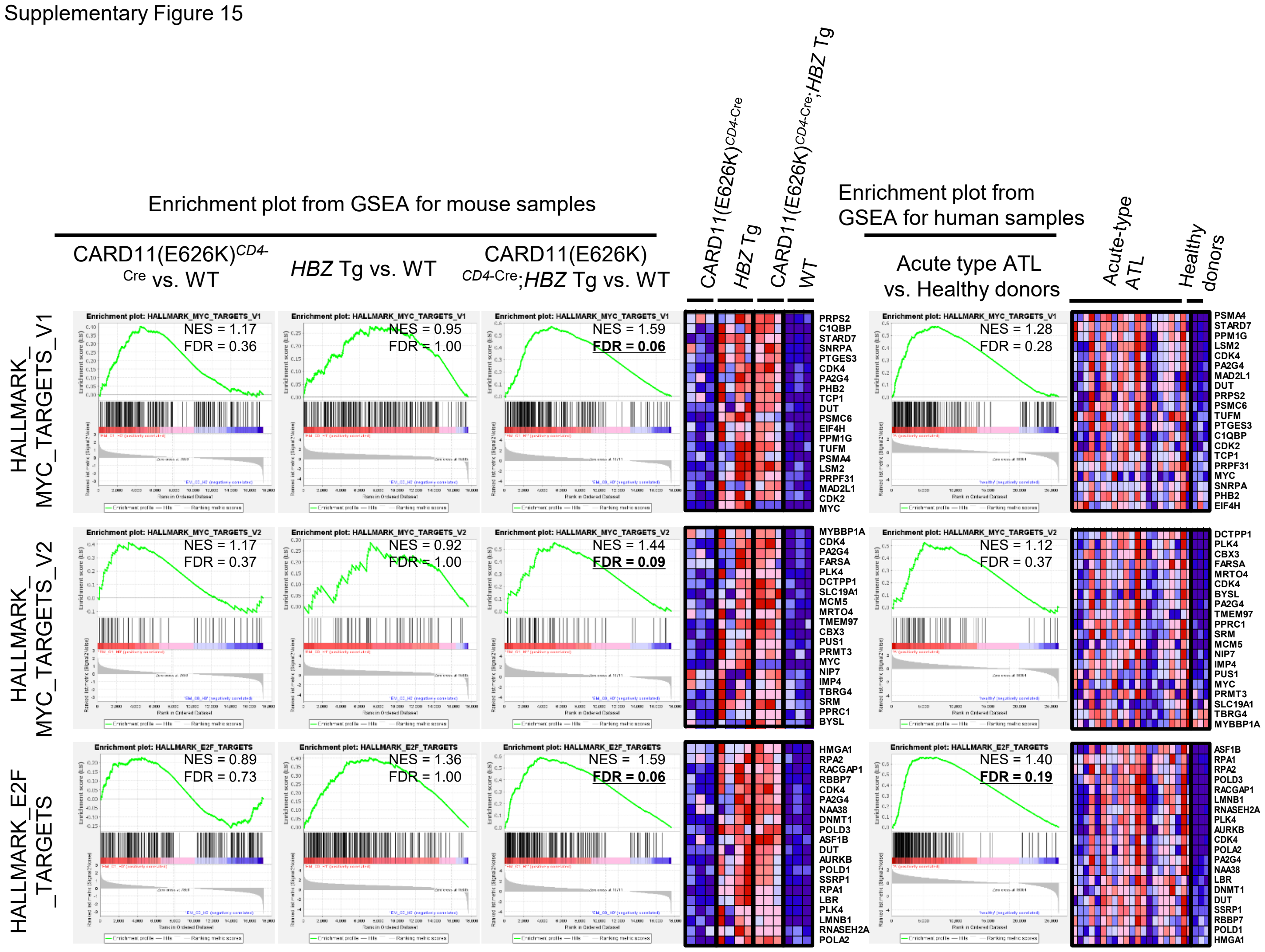

Supplementary Figure 15. Enrichment of proliferation-related gene sets in CARD11(E626K)*CD4*-Cre;*HBZ* Tg mice and human acute-type ATL patient samples.

A cooperative effect between CARD11(E626K)*CD4*-Cre and *HBZ* is observed in proliferation-related gene sets. MYC_TARGETS and E2F_TARGETS were enriched in CARD11(E626K)*CD4*-Cre;*HBZ* Tg samples and human acute-type ATL samples[6].

Supplementary Figure 16

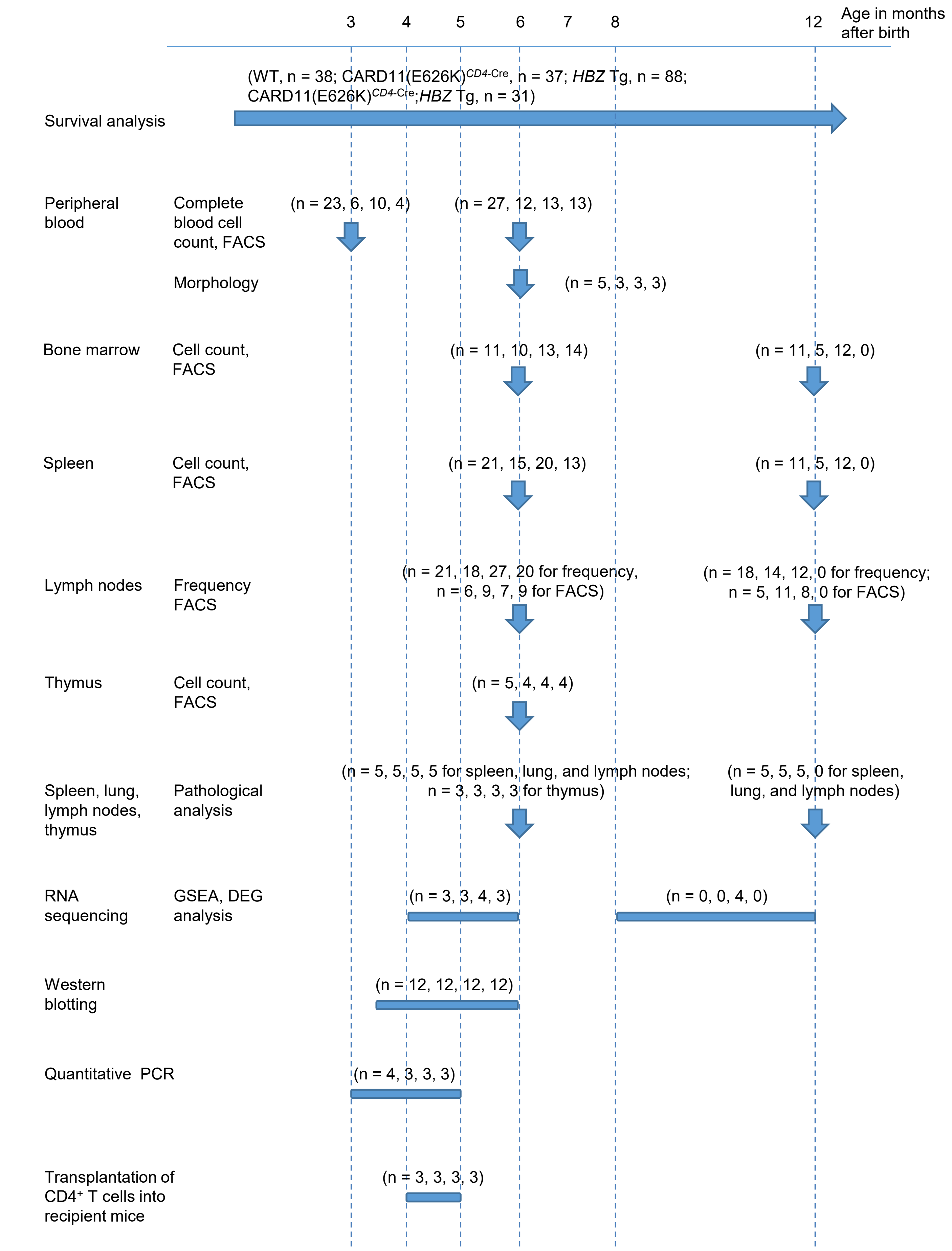

Supplementary Figure 16. Experimental flowchart.

Phenotypic analysis of mutant mice was performed regarding overall survival, peripheral blood, bone marrow, spleen, lymph nodes, thymus, and lung. Functional analysis was performed by RNA sequencing, immunoblotting, and qPCR. Transplantation experiments using splenic $CD4^{+}$ cells from each mouse type were performed using immunocompromised NOD/Shi-scid/IL-$2R\gamma^{null}$ (NOG) mice as the recipients. The timing of analysis is indicated by arrows or horizontal lines, and the numbers of individuals analyzed are stated in the following order: WT, CARD11(E626K)$^{\textit{CD4}\text{-Cre}}$, *HBZ* Tg, and CARD11(E626K)$^{\textit{CD4}\text{-Cre}}$;*HBZ* Tg.

Supplementary Figure 17.

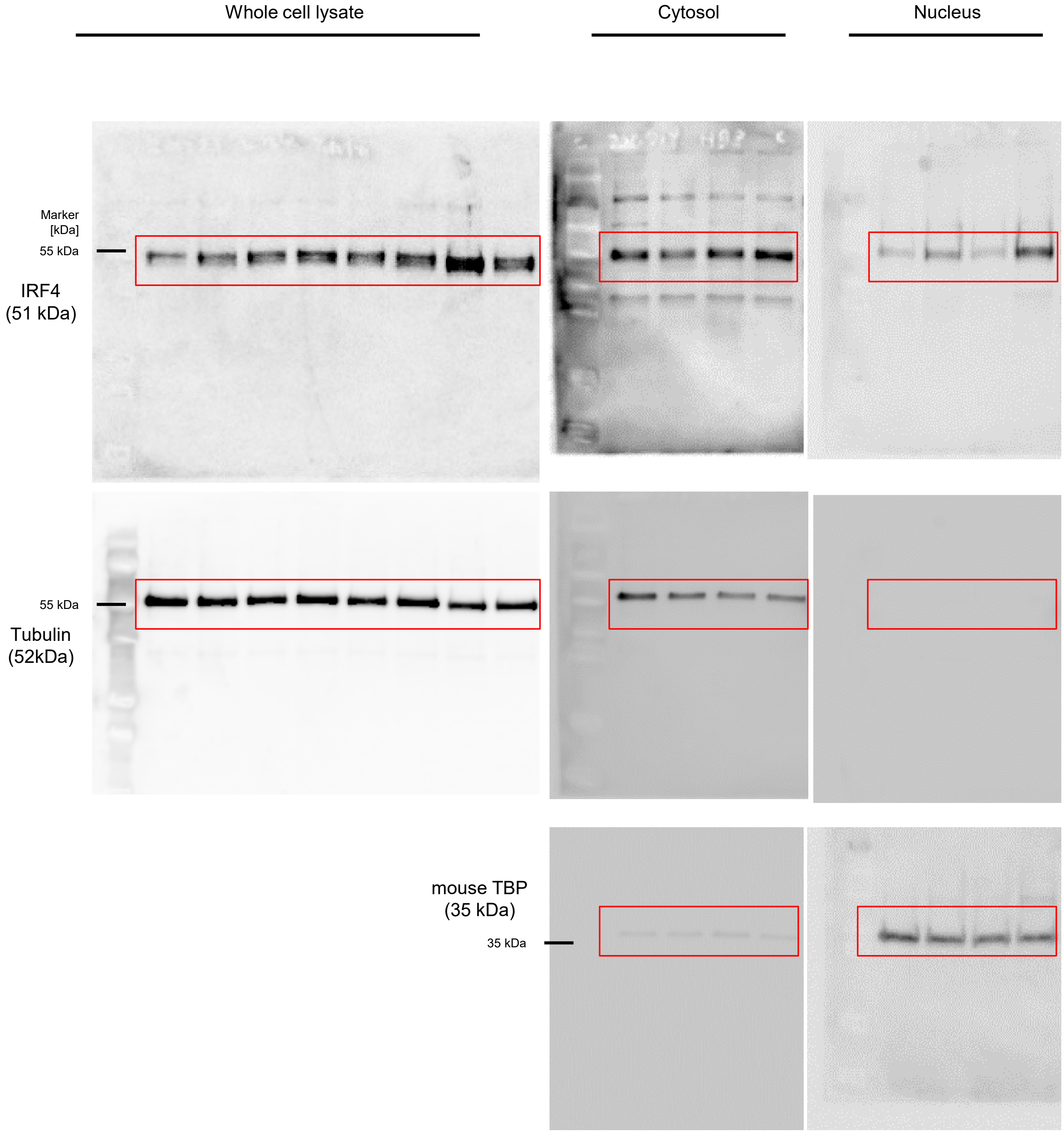

Supplementary Figure 17. Uncropped blots of Figure 9c, d.

Supplementary Figure 18.

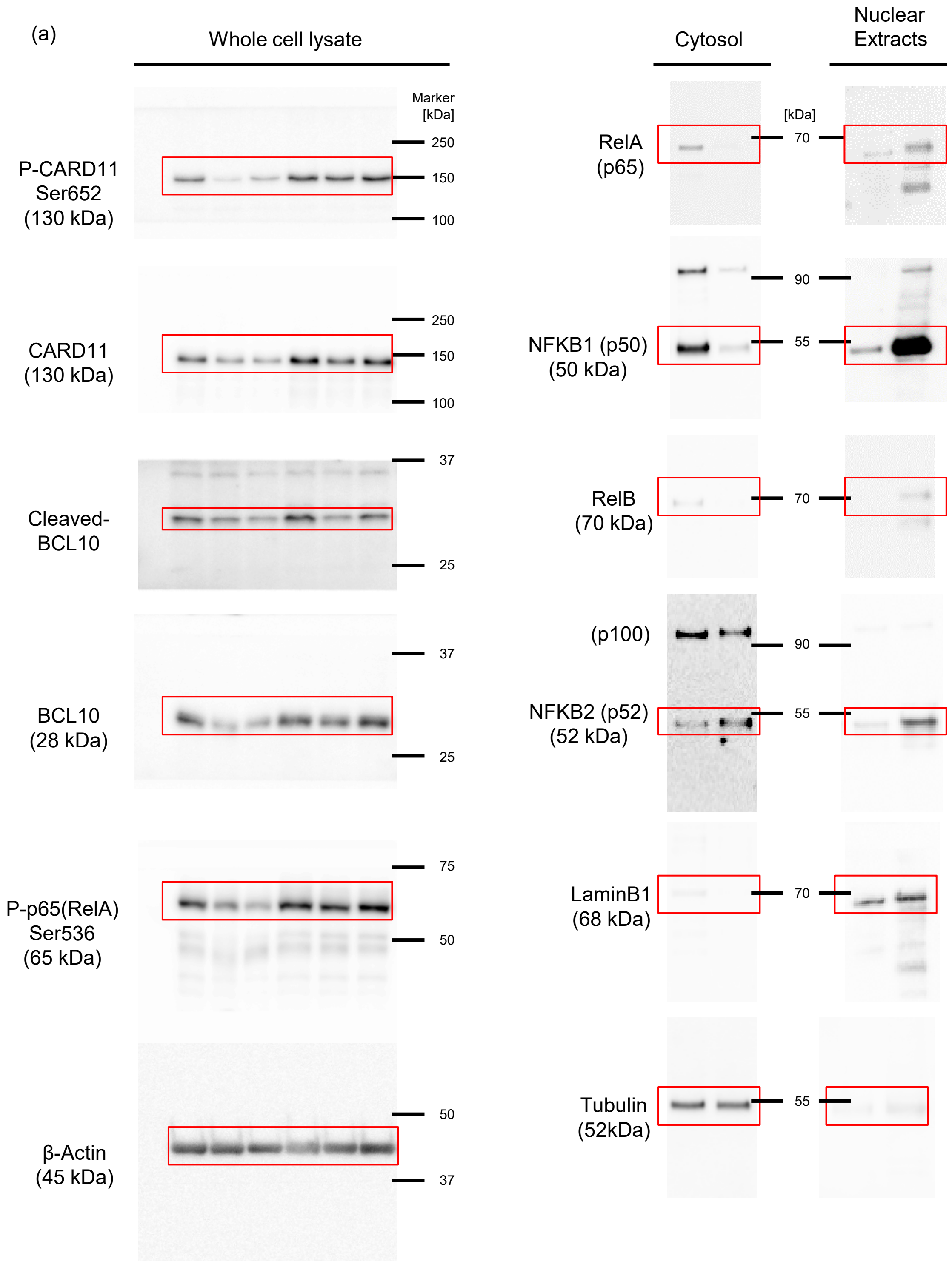

Supplementary Figure 18. Uncropped blots of Supplementary Figure 2

(a) Uncropped blots of Supplementary Figure 2a.

Supplementary Figure 18 (continued)

(b)

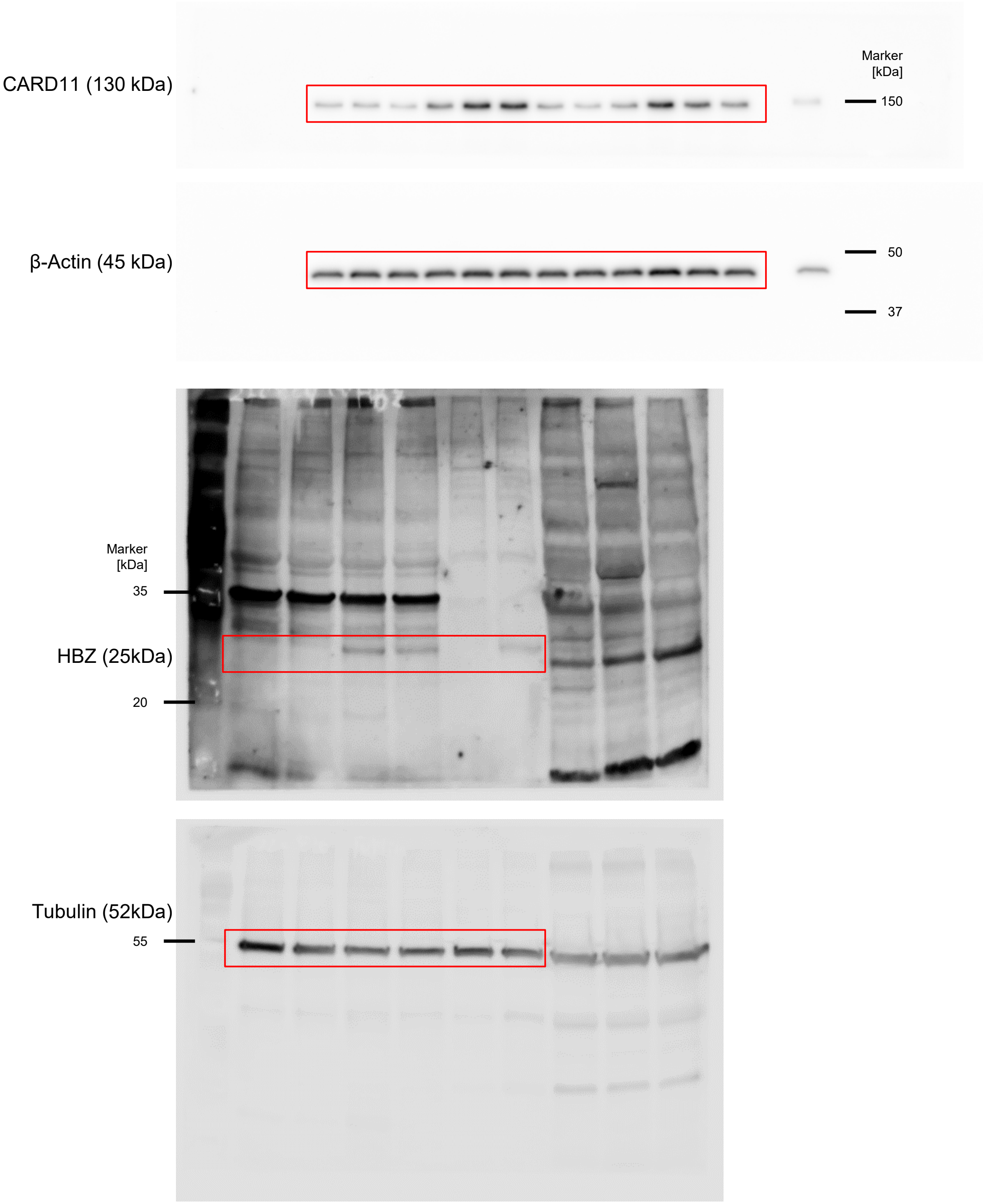

(b) Uncropped blots of Supplementary Figure 2b.

Supplementary Figure 19.

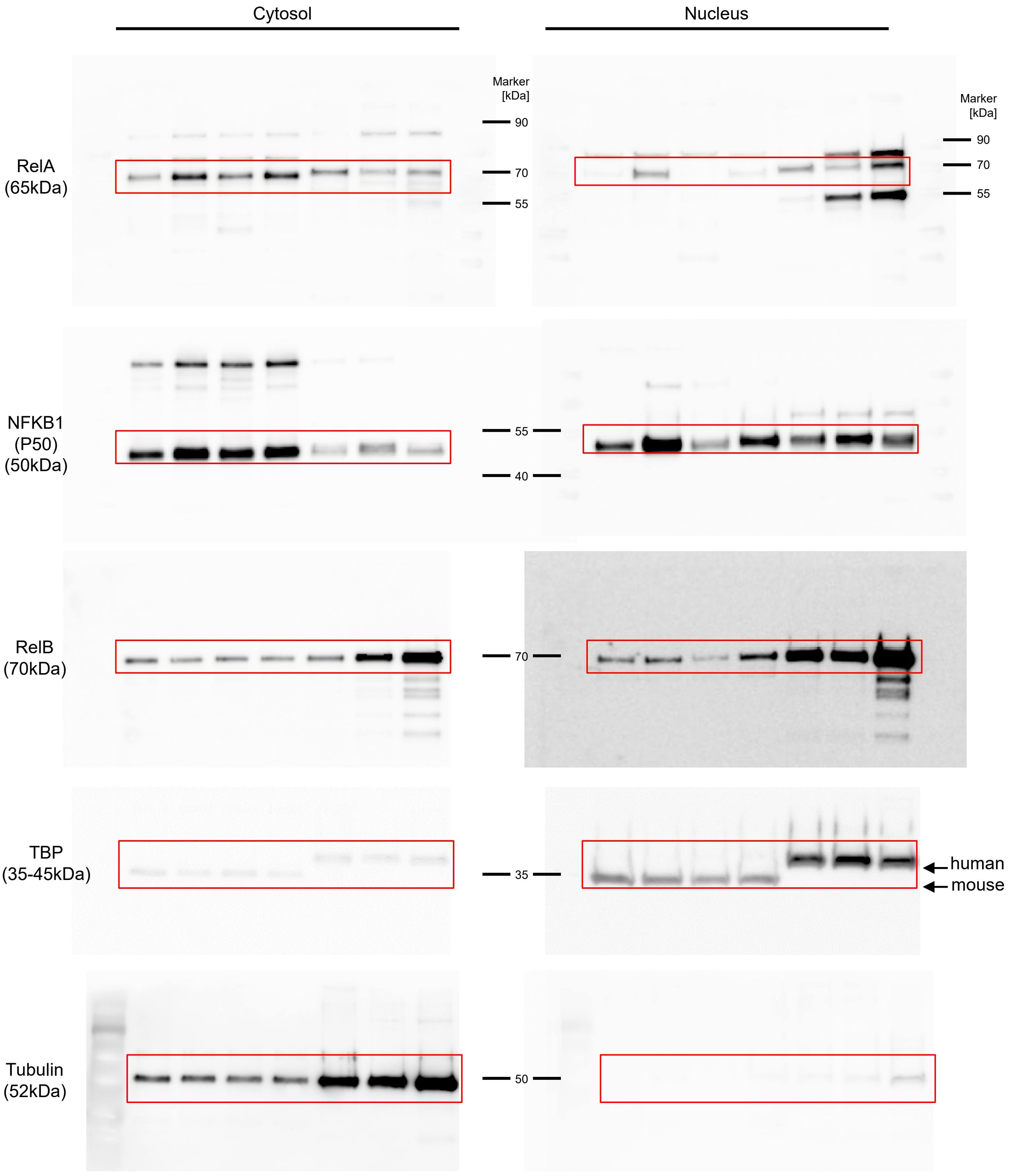

Supplementary Figure 19. Uncropped blots of Supplementary Figure 10.

Supplementary Reference

1 Shide, K. *et al.* Development of ET, primary myelofibrosis and PV in mice expressing JAK2 V617F. *Leukemia* **22**, 87-95, doi:10.1038/sj.leu.2405043 (2008).

2 Bray, N. L., Pimentel, H., Melsted, P. & Pachter, L. Near-optimal probabilistic RNA-seq quantification. *Nat Biotechnol* **34**, 525-527, doi:10.1038/nbt.3519 (2016).

3 Robinson, M. D., McCarthy, D. J. & Smyth, G. K. edgeR: a Bioconductor package for differential expression analysis of digital gene expression data. *Bioinformatics* **26**, 139-140, doi:10.1093/bioinformatics/btp616 (2010).

4 Ge, S. X., Son, E. W. & Yao, R. iDEP: an integrated web application for differential expression and pathway analysis of RNA-Seq data. *BMC Bioinformatics* **19**, 534, doi:10.1186/s12859-018-2486-6 (2018).

5 Subramanian, A. *et al.* Gene set enrichment analysis: a knowledge-based approach for interpreting genome-wide expression profiles. *Proc Natl Acad Sci U S A* **102**, 15545-15550, doi:10.1073/pnas.0506580102 (2005).

6 Liberzon, A. *et al.* The Molecular Signatures Database (MSigDB) hallmark gene set collection. *Cell Syst* **1**, 417-425, doi:10.1016/j.cels.2015.12.004 (2015).

7 Staudt, L. M. Signature DB. *https://lymphochip.nih.gov/signaturedb/*.

8 Chen, E. Y. *et al.* Enrichr: interactive and collaborative HTML5 gene list enrichment analysis tool. *BMC Bioinformatics* **14**, 128, doi:10.1186/1471-2105-14-128 (2013).

9 Zhang, Y., Parmigiani, G. & Johnson, W. E. ComBat-seq: batch effect adjustment for RNA-seq count data. *NAR Genom Bioinform* **2**, lqaa078, doi:10.1093/nargab/lqaa078 (2020).

10 Odqvist, L. *et al.* NIK controls classical and alternative NF-kappaB activation and is necessary for the survival of human T-cell lymphoma cells. *Clin Cancer Res* **19**, 2319-2330, doi:10.1158/1078-0432.CCR-12-3151 (2013).

11 Mitobe, Y., Yasunaga, J., Furuta, R. & Matsuoka, M. HTLV-1 bZIP Factor RNA and Protein Impart Distinct Functions on T-cell Proliferation and Survival. *Cancer Res* **75**, 4143-4152, doi:10.1158/0008-5472.CAN-15-0942 (2015).

12 Higuchi, Y. *et al.* HTLV-1 induces T cell malignancy and inflammation by viral antisense factor-mediated modulation of the cytokine signaling. *Proc Natl Acad Sci U S A* **117**, 13740-13749, doi:10.1073/pnas.1922884117 (2020).

13 Carter, S. L., Eklund, A. C., Kohane, I. S., Harris, L. N. & Szallasi, Z. A signature of chromosomal instability inferred from gene expression profiles predicts clinical outcome in multiple human cancers. *Nat Genet* **38**, 1043-1048, doi:10.1038/ng1861 (2006).

14 Marbach, D. *et al.* Tissue-specific regulatory circuits reveal variable modular perturbations across complex diseases. *Nat Methods* **13**, 366-370, doi:10.1038/nmeth.3799 (2016).

15 Han, H. *et al.* TRRUST v2: an expanded reference database of human and mouse transcriptional regulatory interactions. *Nucleic Acids Res* **46**, D380-D386, doi:10.1093/nar/gkx1013 (2018).
