# Supplementary material for: CARD11 mutation and HBZ expression induce lymphoproliferative disease and adult T-cell leukemia/lymphoma
Source: Commun Biol. 2022 Nov 29;5:1309. doi: 10.1038/s42003-022-04284-x (PMC9709164; doi:10.1038/s42003-022-04284-x)
Supplement: Supplementary file 5 — Reporting Summary [file 42003_2022_4284_MOESM5_ESM.pdf]

nature portfolio

Corresponding author(s): Kazuya Shimoda

Last updated by author(s): Nov 19, 2022

Reporting Summary

Nature Portfolio wishes to improve the reproducibility of the work that we publish. This form provides structure for consistency and transparency in reporting. For further information on Nature Portfolio policies, see our Editorial Policies and the Editorial Policy Checklist.

Statistics

For all statistical analyses, confirm that the following items are present in the figure legend, table legend, main text, or Methods section.

n/a	Confirmed	
☐	☒	The exact sample size (*n*) for each experimental group/condition, given as a discrete number and unit of measurement
☐	☒	A statement on whether measurements were taken from distinct samples or whether the same sample was measured repeatedly
☐	☒	The statistical test(s) used AND whether they are one- or two-sided<br>*Only common tests should be described solely by name; describe more complex techniques in the Methods section.*
☐	☒	A description of all covariates tested
☐	☒	A description of any assumptions or corrections, such as tests of normality and adjustment for multiple comparisons
☒	☐	A full description of the statistical parameters including central tendency (e.g. means) or other basic estimates (e.g. regression coefficient) AND variation (e.g. standard deviation) or associated estimates of uncertainty (e.g. confidence intervals)
☒	☐	For null hypothesis testing, the test statistic (e.g. *F*, *t*, *r*) with confidence intervals, effect sizes, degrees of freedom and *P* value noted<br>*Give P values as exact values whenever suitable.*
☒	☐	For Bayesian analysis, information on the choice of priors and Markov chain Monte Carlo settings
☒	☐	For hierarchical and complex designs, identification of the appropriate level for tests and full reporting of outcomes
☒	☐	Estimates of effect sizes (e.g. Cohen's *d*, Pearson's *r*), indicating how they were calculated

Our web collection on statistics for biologists contains articles on many of the points above.

Software and code

Policy information about availability of computer code

Data collection	RNA sequencing data of mice samples were collected in this study, and Kallisto (version 0.43.1) was used for determining read counts and calculating transcripts per million. RNA sequencing data of mice samples have been deposited in the DNA Data Bank of Japan (DRA015050). RNA sequencing data of human samples were obtained from our previous report in which Genomon pipeline version 2.6.2 was used (EGAS00001001296).
Data analysis	Gene expression analysis was performed with GSEA software (4.1.0), Integrated differential Expression and Pathway Analysis (iDEP) Tools, Enrichr, R version 4.0.3, Molecular Signature Database (MSigDB), and Leukemia/Lymphoma Molecular Profiling Project (LLMPP) collections (SignatureDB).

For manuscripts utilizing custom algorithms or software that are central to the research but not yet described in published literature, software must be made available to editors and reviewers. We strongly encourage code deposition in a community repository (e.g. GitHub). See the Nature Portfolio guidelines for submitting code & software for further information.

Data

Policy information about availability of data

All manuscripts must include a data availability statement. This statement should provide the following information, where applicable:

- Accession codes, unique identifiers, or web links for publicly available datasets
- A description of any restrictions on data availability
- For clinical datasets or third party data, please ensure that the statement adheres to our policy

The cDNA sequences used are listed in Supplementary Table 2. The newly generated plasmid has been deposited in the DNA Data Bank of Japan (LC739268). Gene expression datasets obtained from human samples have been deposited at the European Genome-phenome Archive (EGAD00001001411). Gene expression datasets obtained from mouse samples have been deposited in the DNA Data Bank of Japan (DRA015050). Raw data for graphs are uploaded as Supplementary Data. Uncropped blots are provided as Supplementary Figure 17-19. For data-sharing requests, please contact Kazuya Shimoda (kshimoda@med.miyazaki-u.ac.jp).

Human research participants

Policy information about studies involving human research participants and Sex and Gender in Research.

Reporting on sex and gender	NA
Population characteristics	NA
Recruitment	NA
Ethics oversight	NA

Note that full information on the approval of the study protocol must also be provided in the manuscript.

Field-specific reporting

Please select the one below that is the best fit for your research. If you are not sure, read the appropriate sections before making your selection.

☒ Life sciences ☐ Behavioural & social sciences ☐ Ecological, evolutionary & environmental sciences

For a reference copy of the document with all sections, see nature.com/documents/nr-reporting-summary-flat.pdf

Life sciences study design

All studies must disclose on these points even when the disclosure is negative.

Sample size	Sample size was determined on the basis of data and sample availability.
Data exclusions	No data was excluded.
Replication	In vitro experiments were repeated three times individually and statistics of the measurements were calculated; in the case of only two replications were made, the measurements were directly displayed.
Randomization	Mice were selected for analysis so that there were no differences between groups in the age in weeks at the time of analysis.
Blinding	Blinding was difficult because the purpose of this study was to explore the phenotype of the mice and the cause of death.

Reporting for specific materials, systems and methods

We require information from authors about some types of materials, experimental systems and methods used in many studies. Here, indicate whether each material, system or method listed is relevant to your study. If you are not sure if a list item applies to your research, read the appropriate section before selecting a response.

Materials & experimental systems

n/a	Involved in the study
☐	☒ Antibodies
☐	☒ Eukaryotic cell lines
☒	☐ Palaeontology and archaeology
☐	☒ Animals and other organisms
☒	☐ Clinical data
☒	☐ Dual use research of concern

Methods

n/a	Involved in the study
☒	☐ ChIP-seq
☐	☒ Flow cytometry
☒	☐ MRI-based neuroimaging

Antibodies

Antibodies used

-Antibodies for Western blotting
Rabbit Anti-CARD11 (1D12) Cell Signaling Technology #4435, RRID:AB_2070359
Rabbit Anti-Phospho-CARD11 (Ser652) Cell Signaling Technology #5189, RRID:AB_10621241
Rabbit Anti-Cleaved BCL10 Dr. D. Morishita, Chordia Therapeutics N/A
Rabbit Anti-Bcl10 (C78F1) Cell Signaling Technology #4237, RRID:AB_2228005
Rabbit Anti-Phospho-NF-κB p65 (Ser536) Cell Signaling Technology #3033, RRID:AB_331284
Rabbit Anti-RelA (C22B4) Cell Signaling Technology #4764
Rabbit Anti-RelB (D7D7W) Cell Signaling Technology #10544
Rabbit Anti-NF-κB1 p105/p50 Cell Signaling Technology #13586
Rabbit Anti-NF-κB2 p100/p52 Cell Signaling Technology #4882, RRID:AB_10695537
Rabbit Anti-IRF4 (D9P5H) Cell Signaling Technology #15106
Rabbit Anti-HBZ Dr. Oshima, Tokushima Bunri University Muraki et al. Oncogene 33, 2317–2328 (2014).
Rabbit Anti-β-Actin (13E5) HRP conjugated Cell Signaling Technology #5125, RRID:AB_1903890
Rabbit Anti-TBP Cell Signaling Technology #44059
Rabbit Anti-αTubulin Cell Signaling Technology #2144
Goat Anti-Rabbit IgG HRP linked (secondary antibody) Cell Signaling Technology #7074, RRID:AB_2099233
Sheep Anti-Mouse IgG HRP linked (secondary antibody) GE Healthcare #NA9310V

-Antibodies for flow cytometric analysis and cell sorting
Rat FITC Anti-Mouse/Human CD11b (Mac1) BioLegend #101206, RRID:AB_312789
Rat FITC Anti-Mouse CD41 BioLegend #133903, RRID:AB_1626237
Rat FITC Anti-Mouse CD3 BioLegend #100204, RRID:AB_312661
Rat FITC Anti-Mouse CD4 BioLegend #100406, RRID:AB_312691
Rat PE Anti-Mouse Ly-6G/Ly-6C (Gr-1) BioLegend #108408, RRID:AB_313373
Rat PE Anti-Mouse Ter119 BioLegend #116208, RRID:AB_313709
Rat PE Anti-Mouse CD4 BioLegend #100408, RRID:AB_312693
Rat PE Anti-Mouse/Human CD45R/B220 BioLegend #103208, RRID:AB_312993
Rat PE Anti-Mouse/Human CD44 BioLegend #103008, RRID:AB_312959
Rat APC Anti-Mouse CD8a BioLegend #100712, RRID:AB_312751
Rat APC Anti-Mouse CD62L BioLegend #104412, RRID:AB_313099
Rat APC Anti-Mouse CD25 BioLegend #102012, RRID:AB_312861
Rat Anti-Mouse CD16/CD32 (Mouse BD Fc Block) BD Biosciences #553141, RRID:AB_394656
7-AAD Viability Staining Solution BioLegend #420404

-Antibodies for immunohistochemistry
Rabbit Anti-Mouse/Rat/Human CD3 Abcam #ab16669, RRID:AB_443425
Rat Anti-Mouse CD45R (B220) BD Biosciences #550286, RRID:AB_393581
Rabbit Anti-CD44 proteintech #15675-1-AP, RRID:AB_2076198
Rat Anti-Mouse/Rat FOXP3 eBioscience #14-5773-82
Rabbit Anti-Mouse/Human Ki-67 Abcam #ab15580, RRID:AB_443209
Goat Anti-Rabbit Immunoglobulins Biotinylated Thermo Scientific #31820, RRID:AB_228340

Validation

All antibodies are confirmed to work well on the manufacturer's website.

Eukaryotic cell lines

Policy information about cell lines and Sex and Gender in Research

Cell line source(s)

ATL43 and HPB-ATL2 were kindly provided for our research by Dr. Hasegawa (Nagasaki University). S1T was kindly provided for our research by Dr. Ishitsuka (Kagoshima University).

Authentication

Cell lines used were authenticated for our study.

Mycoplasma contamination

All cell lines used tested negative for mycoplasma contamination using a PCR Mycoplasma test kit (VWR 10181-030).

Commonly misidentified lines
(See ICLAC register)

No commonly misidentified lines were used in this study.

Animals and other research organisms

Policy information about studies involving animals; ARRIVE guidelines recommended for reporting animal research, and Sex and Gender in Research

Laboratory animals	Mouse: C57/BL/6 CD4-Cre Tg (The Jackson lab #022071)<br>Mouse: C57/BL/6 CARD11(E626K)stopFL (This paper)<br>Mouse: C57/BL/6 CARD11(E626K)CD4-Cre (This paper)<br>Mouse: C57/BL/6 HBZ Tg (This paper)<br>Mouse: C57/BL/6 CARD11(E626K)CD4-Cre;HBZ Tg (This paper)
Wild animals	This study didn't involve any wild animals.
Reporting on sex	Mice were homogenous in sex and age prior to grouping.
Field-collected samples	NA
Ethics oversight	Animal studies were performed in accordance with the University of Miyazaki Ethics Committee.

Note that full information on the approval of the study protocol must also be provided in the manuscript.

Flow Cytometry

Plots

Confirm that:

☒ The axis labels state the marker and fluorochrome used (e.g. CD4-FITC).

☒ The axis scales are clearly visible. Include numbers along axes only for bottom left plot of group (a 'group' is an analysis of identical markers).

☒ All plots are contour plots with outliers or pseudocolor plots.

☒ A numerical value for number of cells or percentage (with statistics) is provided.

Methodology

Sample preparation	Single-cell suspensions were collected from the lymph nodes, spleen, and bone marrow of mice and stained with the appropriate surface antibodies after hemolysis.
Instrument	Stained single-cell suspensions were analyzed for general subpopulations of T cells by FACS calibur.
Software	FlowJo
Cell population abundance	Cell sorting was performed by BD FACS Aria II, with a sorting accuracy of >95% for the Tem and Treg fractions.
Gating strategy	Effector/memory T cells were gated as CD4+CD44+CD62L- cells. Regulatory T cells were gated as CD4+CD25+ cells.

☒ Tick this box to confirm that a figure exemplifying the gating strategy is provided in the Supplementary Information.
